# The impact of environmental factors in birdsong acquisition using automated recorders

**DOI:** 10.1002/ece3.3889

**Published:** 2018-04-24

**Authors:** Nirosha Priyadarshani, Isabel Castro, Stephen Marsland

**Affiliations:** ^1^ School of Engineering and Advanced Technology Massey University Palmerston North New Zealand; ^2^ Wildlife and Ecology Group Massey University Palmerston North New Zealand; ^3^ School of Mathematics and Statistics Victoria University of Wellington Wellington New Zealand

**Keywords:** automatic acoustic recorders, birdsong attenuation, ecoacoustics, frequency, transmission height, wind

## Abstract

The use of automatic acoustic recorders is becoming a principal method to survey birds in their natural habitats, as it is relatively noninvasive while still being informative. As with any other sound, birdsong degrades in amplitude, frequency, and temporal structure as it propagates to the recorder through the environment. Knowing how different birdsongs attenuate under different conditions is useful to, for example, develop protocols for deploying acoustic recorders and improve automated detection methods, an essential part of the research field that is becoming known as ecoacoustics. This article presents playback and recapture (record) experiments carried out under different environmental conditions using twenty bird calls from eleven New Zealand bird species in a native forest and an open area, answering five research questions: (1) How does birdsong attenuation differ between forest and open space? (2) What is the relationship between transmission height and birdsong attenuation? (3) How does frequency of birdsong impact the degradation of sound with distance? (4) Is birdsong attenuation different during the night compared to the day? and (5) what is the impact of wind on attenuation? Bird calls are complex sounds; therefore, we have chosen to use them rather than simple tones to ensure that this complexity is not missed in the analysis. The results demonstrate that birdsong transmission was significantly better in the forest than in the open site. During the night, the attenuation was at a minimum in both experimental sites. Transmission height affected the propagation of the songs of many species, particularly the flightless ones. The effect of wind was severe in the open site and attenuated lower frequencies. The reverberations due to reflective surfaces masked higher frequencies (8 kHz) in the forest even at moderate distances. The findings presented here can be applied to develop protocols for passive acoustic monitoring. Even though the attenuation can be generalized to frequency bands, the structure of the birdsong is also important. Selecting a reasonable sampling frequency avoids unnecessary data accumulation because higher frequencies attenuate more in the forest. Even at moderate distances, recorders capture significantly attenuated birdsong, and hence, automated analysis methods for field recordings need to be able to detect and recognize faint birdsong.

## 1 INTRODUCTION

The energy of audio signals reduces as they travel. Thus, the energy of the signal received is always lower than that originally produced. While this acoustic attenuation is relevant to any form of audio processing, it is a particularly important issue in outdoor recordings, where the distances can be long, the sources of noise are significant, and there can be objects between the source and the recorder. In addition, the amount of attenuation is frequency dependent, meaning that the characteristic appearance of the signal can change.

Given the interest in ecoacoustics in general (Gasc, Francomano, Dunning, & Pijanowski, 2017; Sueur & Farina, 2015) and automatic recordings of birdsong for passive acoustic monitoring and surveying in particular, it seems timely to revisit the question of how birdsong attenuates in natural environments. Ecoacoustics considers that sound plays an important role in the ecology of an environment. For example, it can act as a reliable measure of activity in an environment, and it enables this measurement to be carried at large scales in both time and geographical spread. In order to use this information correctly, it is important to understand the methods that are used to perform the measurements, that is, the acoustic recorders. Automatic acoustic recorders capture degraded birdsong, and attenuation makes the analysis of such recordings more difficult than it would otherwise be; it has been suggested by several authors that one cause of poor performance of current automated birdsong recognition methods for natural noisy continuous field recordings is their lower quality compared to manual recordings (Aide et al., 2013; Bardeli et al., 2010; Brighten, 2015; Frommolt & Tauchert, 2014; Jančovič & Köküer, 2015; Jinnai, Boucher, Fukumi, & Taylor, 2012; Potamitis, Ntalampiras, Jahn, & Riede, 2014). In this article, we present the analysis of a set of experiments where we investigated the significance of various factors that could affect how birdsong attenuates with distance in outdoor environments.

There are three principal causes of signal attenuation in atmosphere sound transmission, namely the spherical spreading out of the signal, absorption of the signal by the atmosphere, and the interaction of the signal with other objects, such as the ground, barriers, variations in air pressure, temperature, and humidity. These causes can be combined additively, but their actual modeling is less clear, as they depend upon the way that the sound is produced (as a plane wave, or from a point source, or in‐between (Kinsler & Frey, 1962 and Ingard 1953 for more information).

The difficulty in computing these effects analytically for any real‐world example means that experiments are the most informative way to see the actual effects of acoustic attenuation. This is particularly the case in outdoor environments, where the weather plays a significant role: The effect of wind and ground attenuation is reported as the major sources of sound attenuation when compared to humidity, temperature, fog, and rain (Aylor, 1972; Ingard, 1953). It has been reported that ground attenuation has more influence when the sound source and the receiver are close to the ground (Ingard, 1953; Lemon, Struger, Lechowicz, & Norman, 1981). In addition, the environment also plays a role, with research investigating sound propagation and attenuation with atmospheric transmission, mainly to understand the evolution of acoustic communication and ecological sources of natural selection in birds (Ken, Douglas, & Peter, 1977; Marten & Marler, 1977; Morton, 1975; Richards & Wiley, 1980; Waser & Waser, 1977; Wiley & Richards, 1978).

Habitat type and recording conditions are assumed to have a strong effect on the quality of the bio‐acoustic signals that are recorded with autonomous recorders, and experiments are needed to understand this effect. The aim of this study was to understand how the bird calls recorded degrade with distance in a variety of environmental and weather conditions. This can help in the design of protocols for the use of automatic recorders as well as increasing the accuracy of the analysis of the recorded calls, whether by human or machine.

**Figure 1:**
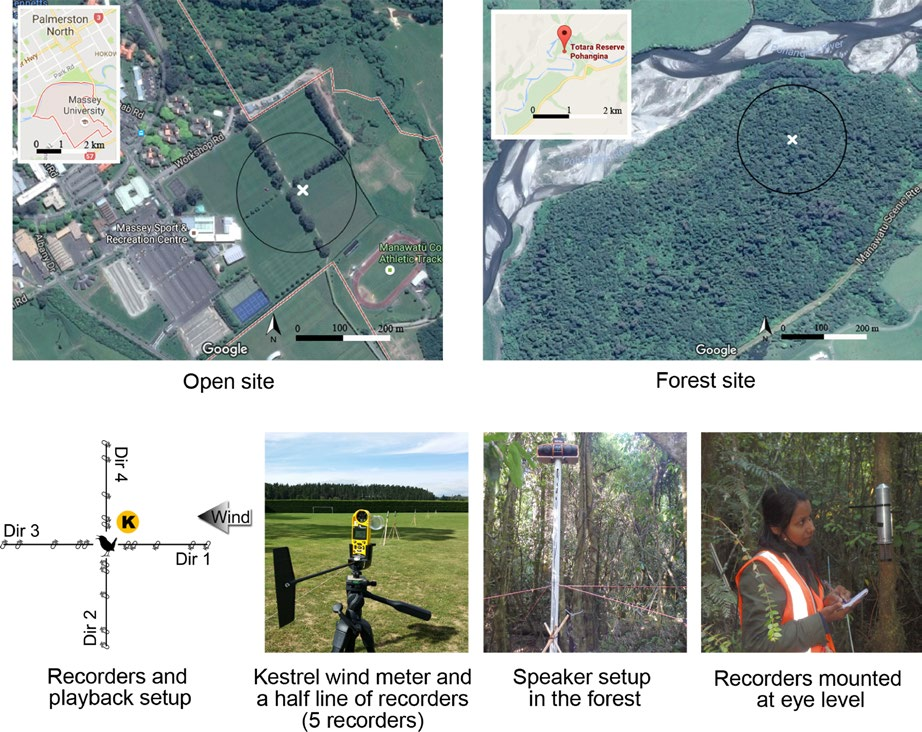
Experimental setup. The location of the speaker is indicated by a white cross in the top two images

Our experiment has a simple playback design: A sequence of bird sounds was broadcast from a speaker, and rings of recorders positioned around the speaker captured and stored the sound. We compared the signal‐to‐noise ratio of the sound files produced by each of the recorders in order to identify which factors affected the quality of the birdsong recorded. The factors tested were (1) open space vs. forest, (2) transmission height (perch height), (3) day vs. night, (4) distance between bird (playback) and recorder, (5) wind direction, and (6) the direction of the bird call in relation to the recorders (Figure 1). An understanding of these factors and how they change the sounds that are recorded is important for the analysis of sounds accumulated in any ecoacoustics or related project.

## 2 MATERIALS AND METHODS

### 2.1 Experimental setup

To observe the effect of the six factors, we set up a playback and recapture experiment with a single sound generator and multiple recorders. Twenty recorders were positioned in five rings around the speaker. The rings were located at 20 m, 25 m, 50 m, 100 m, and 120 m, and the recorders were placed at 0°, 90°, 180°, and 270° with respect to the prevailing wind direction. Effectively, the recorders were positioned along two orthogonal lines that crossed at the speaker location, one of which ran toward and away from the wind direction, and one perpendicular to it (Figure 1). The choice of 20 m was made based on preliminary testing and was sufficiently far away to avoid sound clipping and distortion. The three following distances were simply doubles of each other, while 120 m was a practical limit enforced by the size of the field‐based site. The wind speed and direction were measured using a Kestrel 5500 Weather Meter setup close to the speaker, but with minimal disturbance to the sound transmission (Figure 1). Although the Kestrel meter recorded other environmental conditions such as humidity and temperature, those were not treated as factors in our experiment.

All 20 recorders were automatic acoustic recording units created by the Department of Conservation Electronics Laboratory, Wellington (electronics@doc.govt.nz) recording at 32 kHz sampling frequency. These omnidirectional recorders have −35 dB ± 4 dB sensitivity and 50 Hz to 16 kHz frequency response. We matched recorders with a similar amplitude/frequency response using preliminary playback‐recapture tests using pure sounds (a “click” sound and tonal sounds) generated manually. The recorders were all mounted with the microphone facing the speaker at a height of 1.5 m on wooden stakes (with the support of pegs) in the open field or on trees in the forest (with the support of a metal bracket to hold the recorder; Figure 1).

The speaker was placed on a small platform that was mounted either 0.25 m or 3 m above the ground. These heights were chosen to simulate ground‐based birds, and birds sitting low in the canopy. While it would have been informative to mount the speaker even higher, this was eventually ruled out for practical reasons. Two speakers were used to playback: a boombox GB‐3600 for the very low‐frequency kākāpō and bittern booms and a MiPro MA‐101c for the other calls. The speaker was connected to a Sony PCM‐M10 player via a 5 m long cable.

We selected a wide range of bird sounds from very low frequency to high frequency, and with varying complexity (see the spectrograms in Table 1). A total of 20 different calls/song segments were selected from eleven New Zealand bird species (Table 1), from close‐range recordings (mostly from directional microphones) with minimal noise; those that were not recorded by the authors are in the acknowledgments. All the calls were captured at 44.1 kHz except the Australasian bittern (8 kHz). We matched volume using a combination of subjective analysis (broadcast birdsong were listened by an expert (IC) who indicated when the song sounded as if the bird was calling next to her), and reported measurements of volume; see Table 1. All the values are consistent with those collected for these species using a sound meter by both ourselves and other researchers.

**Table 1:** Details of birds and spectrograms of bird calls used in the experiment. Sound pressure level (SPL) was measured at 1.5 m using a Digitech QM1592 Professional Sound Level Meter following manufacture instructions

Species	Bird group	Time active/habitat type	Call type	Label/SPL (dB)	Spectrogram
North Island brown kiwi (*Apteryx mantelli*)	Flightless ratite	Nocturnal/Forest	Male	bm1 68.7 ± 9.1	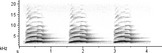
				bm2 72.5 ± 8.2	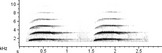
			Female	bf 77.8 ± 5.9	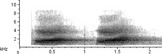
Little spotted kiwi (*Apteryx owenii*)	Flightless ratite	Nocturnal/Forest	Male	lskm1 79.0 ± 5.1	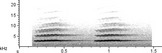
				lskm2 78.9 ± 4.5	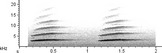
			Female	lskf 80.0 ± 6.7	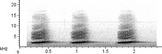
Weka (*Gallirallus australis*)	Flightless rail	Nocturnal/Open/forest	Male/female duet	weka 78.6 ± 8.2	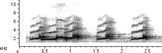
Ruru (*Ninoxi novaesee landiae*)	Owl	Nocturnal/Forest	Morepork	mp 77.1 ± 7.1	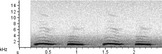
			Trill (low)	trilL 63.8 ± 9.0	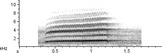
			Trill (high)	trilH 77.4 ± 8.4	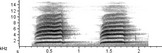
North Island kākā (*Nestor meridionalis*)	Parrot	Diurnal/Forest		kaka 68.6 ± 6.3	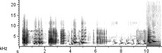
Australasian bittern (*Botaurus poiciloptilus*)	Wetland bird	Crepuscular/Open	Boom	bittern 69.5 ± 6.8	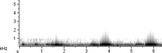
Kākāpō (*Strigops habroptilus*)	Flightless parrot	Nocturnal/Forest	Boom	kBoom 78.3 ± 5.5	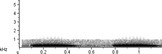
Chinging				kc 69.4 ± 7.8	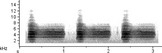
North Island saddleback (*Philesturnus rufusater*)	Passerine	Diurnal/Forest		sad1 69.4 ± 6.3	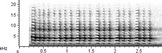
				sad2 67.3 ± 8.2	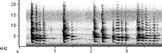
				sad3 58.5 ± 9.0	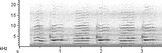
North Island robin (*Petroica longipes*)	Passerine	Diurnal/Forest	Male song	robin 77.8 ± 3.5	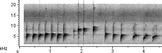
Hihi (*Notiomystis cincta*)	Passerine	Diurnal/Forest		hihi 73.0 ± 5.6	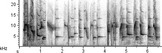
Tūī (*Prosthemadera novaeseelandiae*)	Passerine	Diurnal/Forest		tui 74.8 ± 7.4	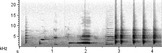

Altogether, the calls, acoustic markers to mark the boundaries of recordings, and intervening silence were about 83 s long. Table 2a summarizes the number of repetitions that occurred within one trial for each bird sound. We repeated the playbacks in order to check the consistency over the four directions and to test for the effect of wind. Therefore, the total length of playbacks for one experiment was approximately 22 min, and this took around one hour to complete including the time to rotate the speaker into the four directions, change the transmission height, switch between the speakers, adjust the volume, and also avoid some evident environment noises such as the calling of wild morepork present in the background (who responded to our playbacks of morepork) and aeroplanes or vehicles passing.

**Table 2:** The trials carried out in the study and playbacks broadcast in each trial. (a) Summary of the playbacks and recaptures one bird sound produced. (b) Summary of the trials carried out. The first column consists of the names given to the trials where the first letter corresponds to the location (open or forest), the second the time of day (day or night), and the third the wind speed (calm, moderate, or windy)

a)		
Transmission direction	Transmission height	Number of repetitions
Dir 1	Low	2
	High	2
Dir 2	Low	2
	High	2
Dir 3	Low	2
	High	2
Dir 4	Low	2
	High	2
Total number of playbacks per bird sound		16
Total re‐recordings per bird sound within one trial		320 (=20 recorders × 16)
Total re‐recordings per bird sound in analysis no wind analysis		1,280 (=4 trials × 320)
Total re‐recordings per bird sound in directionality analysis		320
Total re‐recordings per bird sound in wind speed analysis		1,552 (=5 trials × 320 – 48 missing^a^)

b)				
			Wind observed by Kestrel meter	
Trial	Open/forest	Day/night	Median (Km/hr)	Range (Km/hr)
ODC	Open	Day	3.0 (calm)	0.0–10.0
ODM	Open	Day	7.8 (moderate)	1.2–15.6
ODW	Open	Day	17.5 (windy)	8.4–27.8
ONM	Open	Night	6.6 (moderate)	0.0–14.8
ONC	Open	Night	3.2 (calm)	1.9–7.8
FDC	Forest	Day	0.00 (calm)	0.0–0.0
FNC	Forest	Night	0.00 (calm)	0.0–0.0

a During one trial (ONM: Table 2b), three recorders ran out of battery, resulting in 48 missing data points.

Two relatively flat sites were used to carry out the experiments (Figure 1). The first site was a rugby and soccer field, located at Massey University, divided into four fields by two thin lines of Monterey cypress (*Cupressus macrocarpa*) trees. The distance between the trees was about 3.5 m. For the second site, we selected a native New Zealand forest, the Totara Reserve (40°7′19.1″S 175°51′17.6″E), in the Pohangina Valley near Palmerston North. The reserve is located beside a river and has a road on the other side of the forest (Figure 1). The river was almost dry during the course of experiments, and the recorders did not detect the sound of the river at all. The study site is in the middle of a loop walking track. The selected area is covered by large evergreen trees such as Totara (*Podocarpus totara*), Matai (*Prumnopitys taxifolia*), Rimu (*Dacrydium cupressinum*), and Kahikatea (*Dacrycarpus dacrydioides*). These trees vary between 10 and 50 m in height (with trunks between 50 cm and 2 m in diameter) generating an overlapping canopy with only intermittent view of the sky; the basal area of the trees (cross‐sectional area) is around 60 m^2^ per hectare, and the trees also support a secondary population of creepers (such as supplejack (*Ripogonum scandens*)) and epiphytes (principally kowharawhara (*Astelia solandri*) and kahakaha (*Collospermum hastatum*)).

### 2.2 Data extraction from continuous recordings

Rather than starting and stopping the recorders, we recorded continuously throughout the experiment and postprocessed the complete file to remove the sound between experiments. We used a set of acoustic markers to precisely time stamp the recordings, which was particularly important for the recorders that were further away, and did not detect the birdcalls perfectly. The acoustic markers consisted of a complex pure tone (0.1–44.1 kHz). We used the software Praat to annotate the recordings, by manually identifying the acoustic markers in one recording (a 20 m distant one), and then matching the annotation (TextGrid in Praat) to the other 19 recordings for that trial. This resulted in a text grid with 21 tiers (20 bird sounds and the noise component used to measure the dependent variable). All the recaptures of each bird sound (captured by 20 recorders) were segmented and stored separately, and then, the intensity of the signal measured using Praat scripts.

### 2.3 Dependent variable and covariates

We have chosen one simple measure that captures the most important part of acoustic attenuation, namely the signal‐to‐noise ratio (SNR). This measures the ratio of the power of the signal recorded and the power of the noise. Thus, a large value indicates a clearer signal.

There are two challenges with using this concept: Neither the pure signal nor the pure noise is generally known. We could have compared the broadcast signal and the received one, but this would not include noise added by the speaker. It would also require perfect temporal lining‐up of the two sounds. We therefore used a variant of the SNR, which we term SnNR (Priyadarshani, Marsland, Castro, & Punchihewa, 2016):

(1) $$\text{SnNR}=\frac{S+N}{N},$$

where *S*+*N* is the intensity of the recorded bird sound, and *N* is the intensity of the background noise at the recorder. To measure *N*, four 10 s sections that did not contain audio signal (in‐between the playbacks) were selected and the power in those segments averaged for each recording. As a consequence of our interest of selecting actual bird calls rather than synthetic sounds, our playback sounds include some level of noise despite the fact that the recordings were achieved at a close range with directional microphones (Table 1). In order to take this point into account, we computed the SnNR of the sounds broadcast and the SnNR of the sounds recaptured (for two examples, tui and mp), then we treated the ratio of these SnNRs as our second measure. As the preliminary analysis confirmed that both measures yield similar results, we used the initial measure throughout the analysis. Accordingly, in the following analyses, SnNR is our dependent variable. The covariates we manipulated were habitat (open/forest), time of the day (day/night), transmission height (low = 0.25, high = 3 m), distance (20 m, 25 m, 50 m, 100 m, 120 m), recorder direction (Dir1–4), and wind speed (calm, moderate, windy).

### 2.4 Statistical method

We explored the predictive value of each of the factors on individual bird sounds using generalized linear models (GLM). The observations were independent; therefore, the assumption of GLM was satisfied. Prior analysis of the data confirmed that the distribution of data followed a gamma distribution, being skewed toward larger values. GLM requires some transformation of Xβ (Equation (2)), the linear predictor of covariates (*X*), to guarantee additivity. The coefficients of the linear predictor are contained in β. The link function, *g*, defines this relationship between the random component (probability distribution of the response variable) and the systematic component (the explanatory variables in the model):

(2) $$E\left[Y\right]=g^{-1}\left(X\beta \right)$$

A comparison of the log and the power functions showed that the inverse link function was the best fit with the data (Table 3). Pearson's chi‐squared method was used as the scale parameter method (Anderson et al., 2004), with a hybrid of Fisher and Newton–Raphson methods. *p* value correction (with sequential Sidak) was carried out to avoid type I errors (Abdi, 2007) because we performed multiple tests of mean effect. Both forward and backward selections were employed to find the optimum model, discarding insignificant effects (Table 3; see Table S4.1 for details of the final models). Even though in some cases the deviance of the final model (Model IV) was slightly larger (compared to Model III; Table 3), we used Model IV as the optimum model because model III had insignificant factors/interactions while model IV had only significant factors/interactions.

**Table 3:** GLM model development—goodness of fit in each model was measured using the deviance^a^

	Deviance			
Call example	Model I (log link)	Model II (inverse link)	Model III (after forward/backward)	Model IV (after forward/backward)
bf	6.680	5.939	3.557	3.564
bm1	5.825	5.675	4.064	4.086
bm2	6.227	5.969	3.781	3.796
lskf	6.216	5.970	4.056	4.080
lskm1	5.725	5.512	3.849	3.850
lskm2	5.940	5.815	4.326	4.330
mp	6.042	5.568	3.025	3.032
trilH	6.248	6.033	4.294	4.299
trilL	5.151	5.093	4.426	4.622
bittern	4.266	4.269	4.026	4.048
kBoom	4.598	4.596	4.044	4.172
kc	5.442	5.423	5.001	–
weka	6.741	6.307	3.775	3.788
kaka	5.512	5.387	4.449	4.455
hihi	6.239	6.222	5.891	5.944
robin	6.939	6.879	6.149	6.191
tui	5.443	5.350	4.224	–
sad1	5.259	5.187	3.358	3.384
sad2	5.652	5.602	4.095	4.158
sad3	5.044	4.975	3.283	3.481

a Information criterion is smaller is better.

For each bird sound, three GLM models were built using different subsets of data (Figure S1–S3). The first analysis (“No wind analysis”) comprised the four trials carried out when the wind was calm (ODC, ONC, FDC, and FNC) as there were most of those, and compared the other effects. There was no missing data in this set; therefore, the total number of data points per bird sound was 1,280 (Table 2a). To compare the effects, we used plots of the estimated marginal means under the GLM models, rather than the means of the raw data, as they take into account the effect of the other variables. The second analysis (“Directionality analysis”) used data from the same four trials used in the first analysis, but the speaker direction was fixed. This enabled us to investigate the effects caused by the fact that birdcalls are directional. Excluding speaker direction reduced the data size to 320 recordings per bird sound. The third analysis (“wind speed analysis”) used data from the trials carried out in different wind speeds in the open field (ODC, ODM, ODW, ONC, and ONM) to study the effect of wind direction explicitly. For each model, we looked at the effect of each factor separately and the effect of all possible interactions. The statistical analyses were carried out using SPSS^®^ version 22, and 99% confidence intervals (α = 0.01) were used.

## 3 RESULTS

### 3.1 No wind analysis

#### 3.1.1 Day vs night

There was no significant difference in the SnNR between day and night for passerine birds except one call example of saddleback (sad1; Table 4, Table S4.3, and Figure S4.1). Although the SnNR varied between 1.02 and 1.14 in the daytime and from 0.99 to 1.22 in the night for different bird sounds, the trends of SnNR were lower during the day than night for each bird sound tested. Bittern and kākāpō booms consistently followed the opposite pattern (their SnNR was significantly higher during the day). It was evident from these results that the sound transmission of nocturnal birds was significantly better during the night compared to the day.

**Table 4:** No wind analysis: The main effects found in each model . Note that this table was generated from 20 individual GLM statistical tests (for each bird sound example). *df* = degrees of freedom. In bold, data for factors that were in significant

Bird sound	Model effect	(Intercept)	Day/night	Open/forest	Height	Recorder direction	Bird direction	Distance
	*df*	1	1	1	1	3	3	4
bf	Wald ×2	258,020	337	1,041	322	43	3	3,446
	*p* value	.000	.000	.000	.000	.000	**.378**	.000
bml	Wald ×2	286,412	13	258	0	27	7	1,187
	*p* value	.000	.000	.000	**.481**	.000	**.085**	.000
bm2	Wald ×2	270,971	26	359	10	20	2	1,577
	*p* value	.000	.000	.000	.002	.000	**.513**	.000
lskf	Wald ×2	270,572	35	435	13	12	1	1,383
	*p* value	.000	.000	.000	.000	.008	**.745**	.000
lskml	Wald ×2	290,832	59	328	0	27	6	1,790
	*p* value	.000	.000	.000	**.744**	.000	**.091**	.000
lskm2	Wald ×2	279,342	11	222	0	16	4	1,146
	*p* value	.000	.001	.000	**.632**	.001	**.265**	.000
mp	Wald ×2	278,838	52	930	114	18	0	1,653
	*p* value	.000	.000	.000	.000	.000	**.826**	.000
trilH	Wald ×2	272,516	19	330	22	9	4	1,153
	*p* value	.000	.000	.000	.000	**.026**	**.224**	.000
trilL	Wald ×2	318,858	0	199	7	5	5	520
	*p* value	.000	**.651**	.000	**.010**	**.174**	**.184**	.000
bittern	Wald ×2	380,904	58	0	2	21	10	200
	*p* value	.000	.000	**.865**	**.212**	.000	**.021**	.000
kBoom	Wald ×2	347,271	63	3	2	16	19	125
	*p* value	.000	.000	**.096**	**.188**	.001	.000	.000
kc	Wald ×2	301,043	0	124	22	5	3	440
	*p* value	.000	**.905**	.000	.000	**.189**	**.356**	.000
weka	Wald ×2	253,858	85	499	50	37	7	2,047
	*p* value	.000	.000	.000	.000	.000	**.089**	.000
kaka	Wald ×2	302,111	15	354	0	10	0	917
	*p* value	.000	.000	.000	**.506**	**.020**	**.944**	.000
hihi	Wald ×2	262,249	2	104	27	0	2	366
	*p* value	.000	**.142**	.000	.000	**.922**	**.606**	.000
robin	Wald ×2	239,984	1	100	6	2	3	617
	*p* value	.000	**.259**	.000	.014	.**560**	**.408**	.000
tui	Wald ×2	303,147	0	240	1	12	3	770
	*p* value	.000	**.961**	.000	**.248**	.008	**.351**	.000
sad1	Wald ×2	314,182	16	149	0	13	16	910
	*p* value	.000	.000	.000	**.515**	.004	.001	.000
sad2	Wald ×2	294,047	3	115	0.10	20	17	657
	*p* value	.000	**.113**	.000	**.751**	.000	.001	.000
sad3	Wald ×2	329,656	2	111	6	32	22	930
	*p* value	.000	**.120**	.000	**.019**	.000	.000	.000

#### 3.1.2 Open vs forest

SnNR was higher in the forest compared to the open area except for the very low‐frequency booms of kākāpō and bitterns, which transmitted equally at both sites (Table 4). The Kestrel meter did not detect any wind in the middle of the forest (close to the speaker) under the canopy, and the actual wind during the trials was always around 10 km/hr.

**Figure 2:**
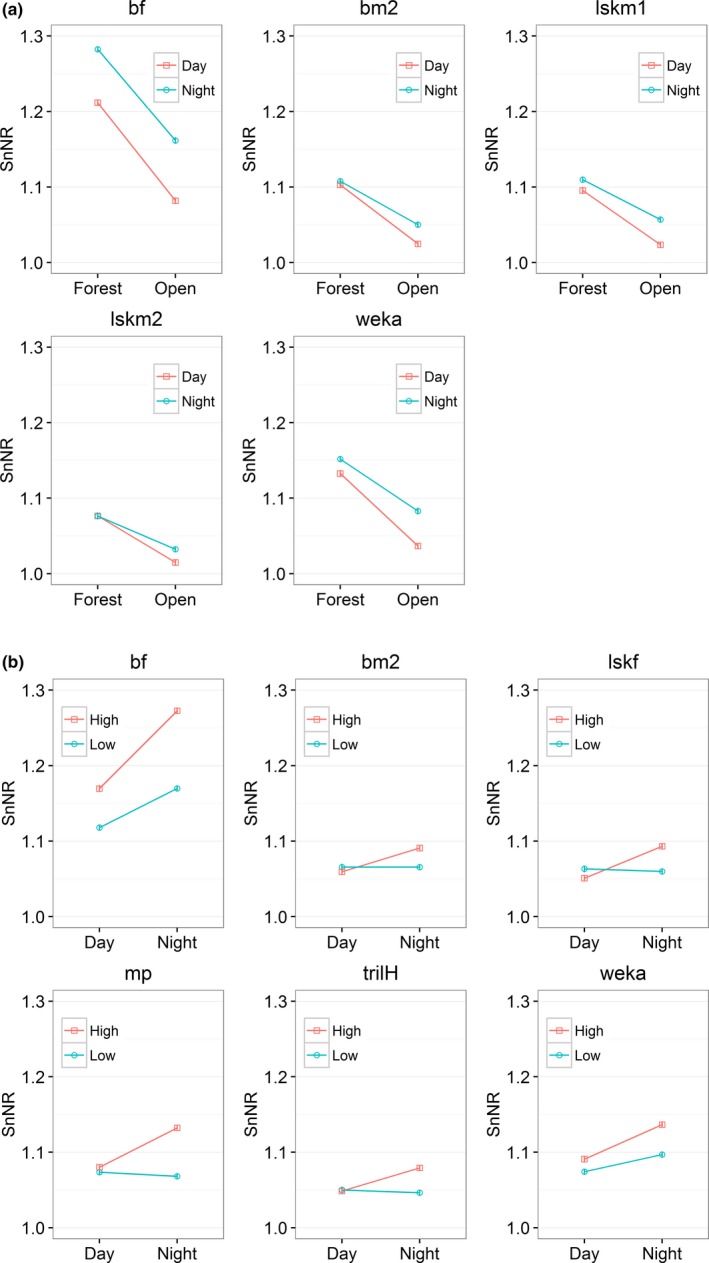
Interaction effect between time of the call and site/transmission height. Bars represent standard errors. The lines were added to showcase the trend for each test result. bf = brown kiwi female, bm2 = brown kiwi male example 2, lskm1 = little spotted kiwi male example 1, and lskm2 = little spotted kiwi male example 2. lskf = little spotted kiwi female, mp = more‐pork sound of morepork, and trilH = trill (high) sound of morepork. (a) Estimated marginal means of SnNR for interaction effect of site and time of the call. The figure was generated from 5 individual GLMs (for each bird sound example). (b) Estimated marginal means of SnNR for interaction effect of the transmission height and the time of the call. The figure was generated from 6 individual GLMs (for each bird sound example)

There was a significant interaction effect of site and the time of day on the two species of kiwis and weka (Figure 2a and Table S4.7). SnNR was highest when these nocturnal species vocalized in the forest at night and lowest when they vocalize in the open area during the day. The average SnNR for three kiwi examples and weka was approximately similar in the forest despite the time of day, but significantly different from each other in the open site (Figure 2a).

#### 3.1.3 Transmission height

**Figure 3:**
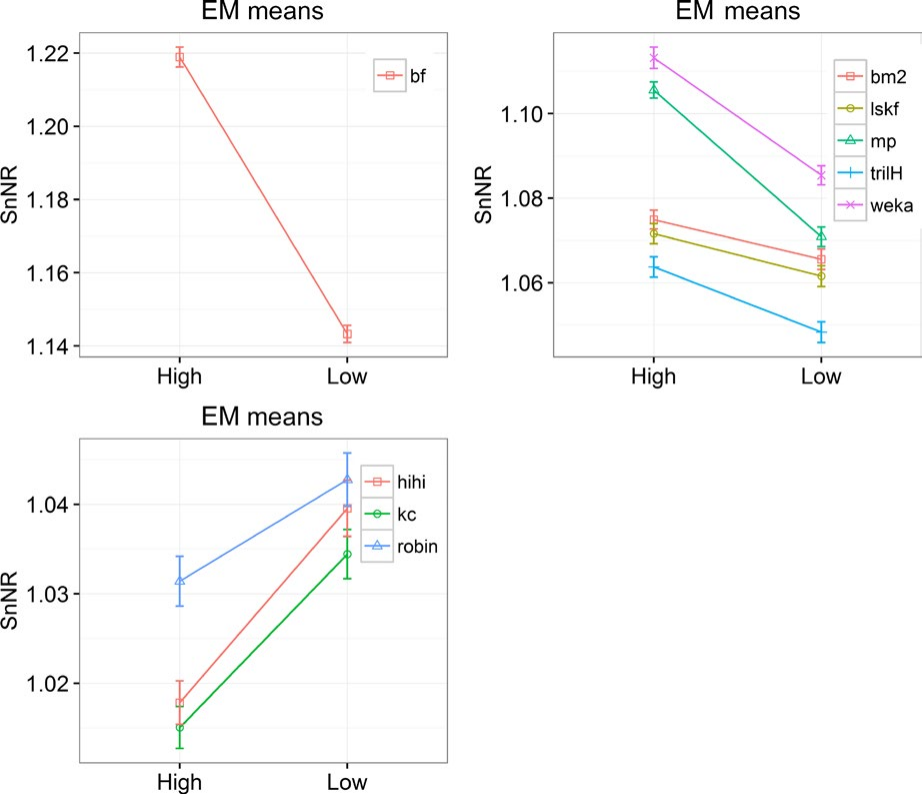
Estimated marginal means of SnNR for two transmission heights. Bars represent standard errors. Note that this figure was generated from 9 individual GLMs (for each bird sound example) and the lines were added to showcase the trend for each test result. bf=brown kiwi female, bm2 = brown kiwi male example 2, kc = kākāpō chinging, and lskf = little spotted kiwi female, mp = more‐pork sound of morepork, and trilH = trill (high) sound of morepork

Transmission height had a significant effect on some vocalizations of the ground‐dwelling species considered (Figure 3). The sound transmission was better at 3 m height for two kiwi females and weka. Hihi and robin sounds were better heard when the speaker was close to the ground.

**Figure 4:**
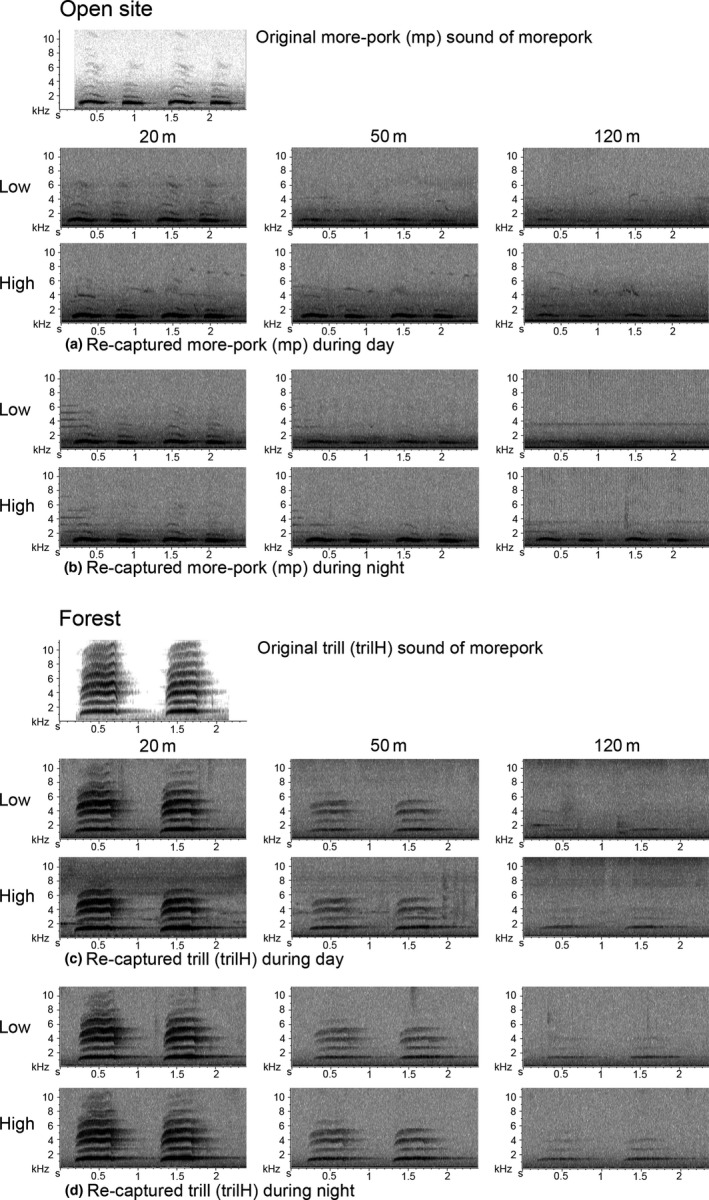
Recaptured sounds from morepork broadcasts of more‐pork (mp) and trill (trilH). In all cases the speaker was facing the recorders and the wind was calm

Kākāpō chinging transmitted better close to the ground, particularly in the open field (Figure S4.3), and morepork sounds were better heard when broadcast higher. Spectrogram inspection of recaptured morepork sounds also confirmed that their attenuation was higher when the sound was transmitted close to the ground both in the open site and the forest (Figure 4). The best transmission was always during the night when the sound was broadcast from the “high” transmission height (Figure 2b).

For the sounds of some species, there were interaction effects between the transmission height, and the site and the time of the day (Table S4.8‐S4.9, and Figure 2b). When these bird sounds were transmitted at high transmission height, sound transmission was markedly better during the night compared to the day (Figure 2b). Overall, high interaction effect between the transmission height and the time of call compared to the interaction effect between the transmission height and the site was evident.

#### 3.1.4 Distance

**Figure 5:**
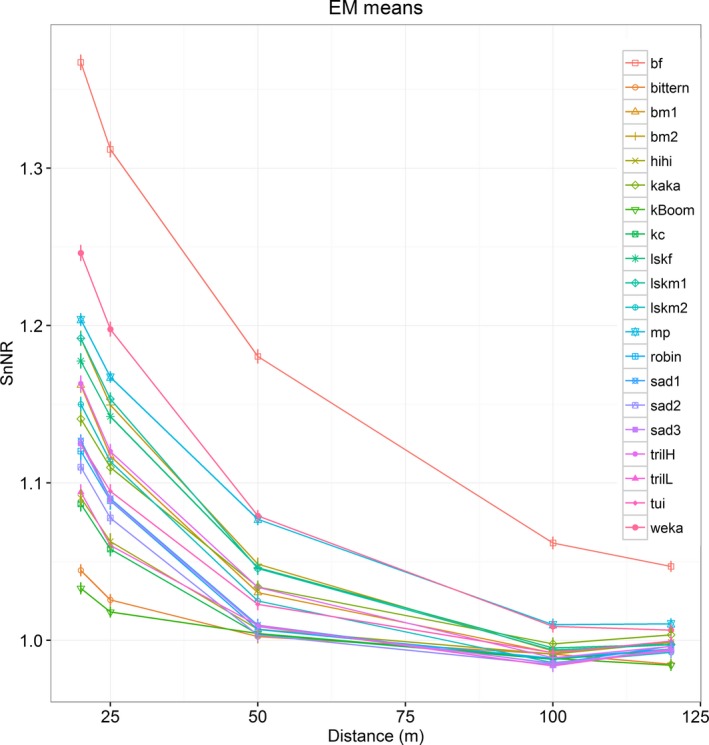
Estimated marginal means of SnNR against distance. Bars represent standard errors. Note that this figure was generated from 20 individual GLMs (for each bird sound example) and the lines were added to showcase the trend for each test result. bf=brown kiwi female, bm1 = brown kiwi male example 1, bm2 = brown kiwi male example 2, kBoom = kākāpō boom, kc = kākāpō chinging, lskf = little spotted kiwi female, lskm1 = little spotted kiwi male example 1, lskm2 = little spotted kiwi male example 2, mp = more‐pork sound of morepork, sad1 = saddleback example 1, sad2 = saddleback example 2, sad3 = saddleback example 3, trilH = trill (high) sound of morepork, and trilL = trill (low) sound of morepork

SnNR decreased significantly (Table S4.5‐S4.6) with distance (Figure 5). There was a significant interaction effect (Table S4.10) between the habitat and the distance to the broadcast song for all bird sounds except the booms. Recordings in the forest exhibited higher SnNR than those in the open site, and the difference was highest at short distance and decreased with increasing distance. The difference was minimal after 100 m.

**Figure 6:**
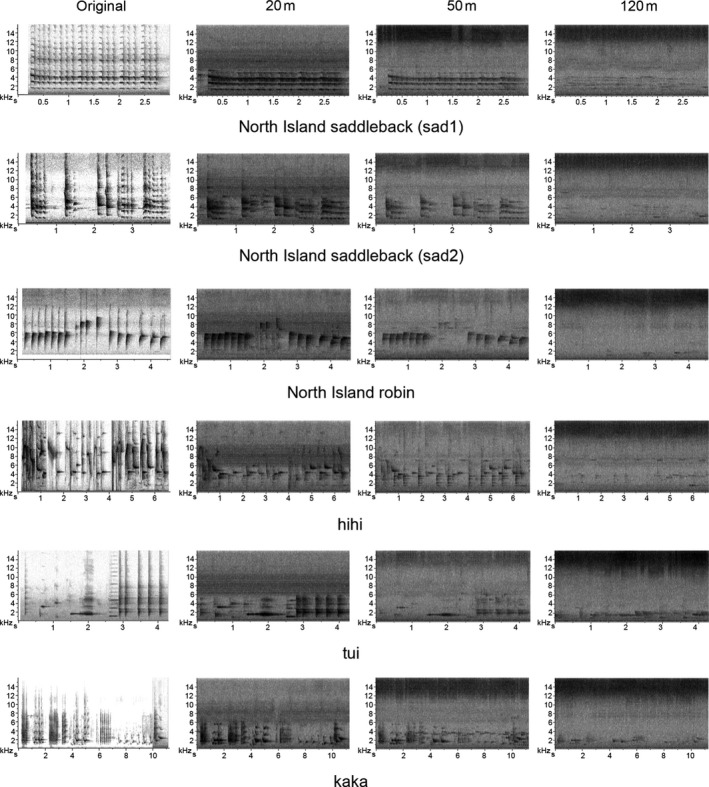
Recaptured birdsong that were transmitted from 3 m to the ground in the forest during the day. Speaker was facing the recorders (20 m, 50 m, and 120 m) positioned in one direction

We noted attenuation of birdsong with increasing distance to the recorder in the forest. Even at the relative short distance of 20 m, frequencies beyond 6 to 8 kHz were exceptionally attenuated (Figures 4 and 6). However, at 50 m calls still carried most of the frequency components they had at 20 m, but with less energy. The recorder at 120 m perceived only the kiwi and morepork calls.

### 3.2 Directionality analysis

**Figure 7:**
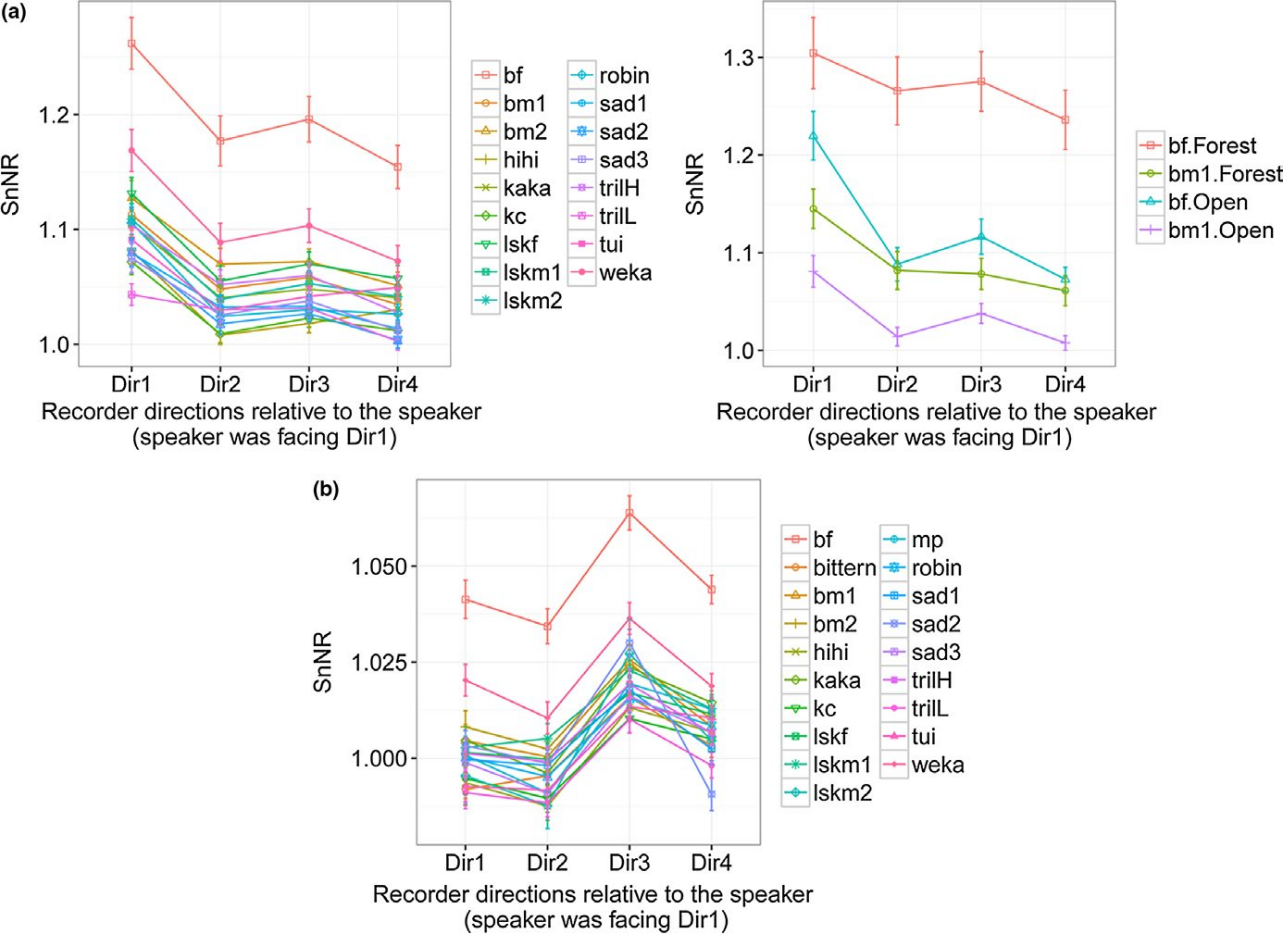
The effect of sound transmission direction and wind on signal quality (SnNR). Dir1–4 are as given in Figure 1. (a) The change of the SnNR when the speaker was facing Dir1 (Directionality Analysis). Estimated marginal means of SnNR against the four recorder directions on the left (generated from 17 individual GLMs) and estimated marginal means of SnNR against the four recorder directions in open vs forest for a male and a female brown kiwi example on the right (bf and bm1; generated from 2 individual GLMs). (b) Estimated marginal means of SnNR against the four recorder directions when the bird calls to all four directions equally (Directionality Analysis; generated from 19 individual GLMs). The dataset includes `calm’, `moderate’, and `windy’ data in the open site

Directionality analysis investigated the effect of the speaker direction on the quality of the recordings collected to reflect the fact that birdcalls are directional. This variable always had a significant effect on the quality (SnNR) of the recordings except in the case of low‐frequency kākāpō boom (kBoom) and more‐pork (mp) sounds (Table S4.11). As we expected, when the speaker was facing the recorder (Dir1), the SnNR was higher (left figure in Figure 7a). Those behind the speaker (Dir3) also had better SnNR; further analysis showed this is due to the effect of the wind at the open site (right figure in Figure 7a).

### 3.3 Wind speed analysis

The influence of wind was more prominent in the open space than the closed forest habitat. Therefore, wind speed analysis focused on five trials carried out under different wind conditions, “calm” (<4 km/hr), “moderate” (6–8 km/hr), and “windy” (>15 km/hr) in the open site (Table 2b). The overall highest SnNR was gained by the recorders in the line away from the wind direction (Dir3 in Figure 7b; Figure 1). The relative direction of the speaker to the recorder was not significant in the case of kākāpō booming (Table S4.12).

**Figure 8:**
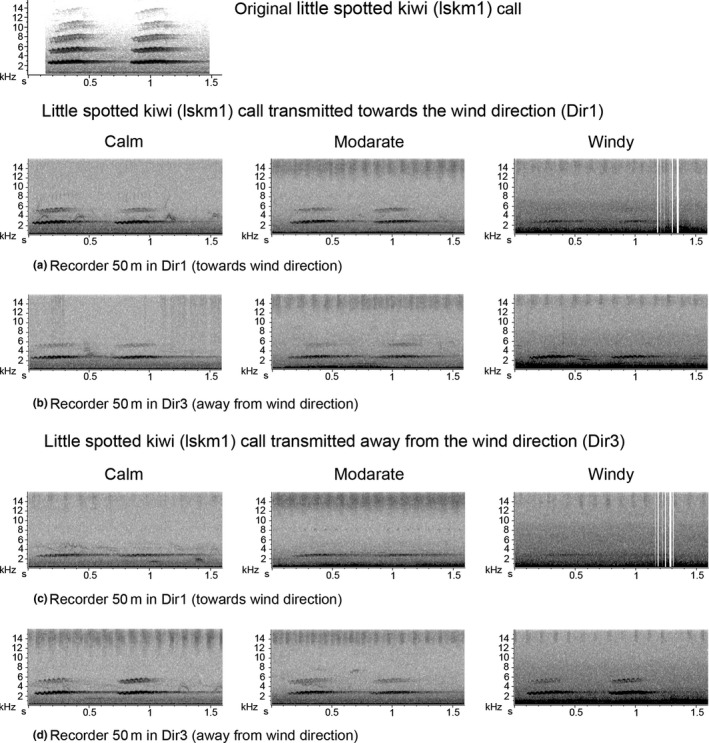
Recaptured little spotted kiwi call (lskm1) transmitted close to the ground in the open site under different wind levels when the speaker was facing the wind direction and away from the wind direction. The vertical lines in the spectrograms in first and third rows in the third column are due to gusts of wind while the high‐frequency noise visible as dark line shadows in most of the spectrograms is possibly due to the noise of a watering machine. Compare to the original sound to discriminate any noise from the bird sound

Regardless of speaker direction, the downwind recorders (Dir3) captured the bird sounds better, particularly when the wind was strong [e.g., Figure 8 for male little spotted kiwi call (lskm1)]. In contrast, sounds were lost in the spectrograms from the recorder positioned upwind (Dir1). The wind intensity significantly reduced the SnNR of the recordings for all the bird sounds (Table S4.13 and Figure S4.4). However, kākāpō and bittern booms did not show a significant difference at “moderate” and “windy” levels while kākāpō chinging (kc), and the trill sound of morepork (trilL) did not show a significant difference at “calm” and “moderate” levels (Table S4.13).

## 4 DISCUSSION

Natural environments make it extremely difficult to predict sound attenuation reliably; in real outdoor conditions, distance from the source is only one factor among many. In this article, we have used real birdsong recordings to examine what omnidirectional autonomous field recorders record in varying environmental conditions. In future work, we will use the results of these experiments to devise protocols for the use of automatic acoustic recorders to survey birds.

There is a variety of possible measurements that can be used to identify degradation of the audio signal. For example, the loss of higher harmonics can be observed as the recorder and player get further apart. A sound level meter, sometimes referred to as sound pressure level meter, can be used to measure the intensity of sound at the receiver when the transmitted signals are pure tones (Marten & Marler, 1977), but we transmitted real bird sounds. Therefore, for this article, we used a variation of SNR as the song measurement.

Amplitude fluctuations and reverberations were studied by broadcasting pure tones by Richards and Wiley (1980) in an experiment similar to ours. They observed that higher frequencies usually attenuate more with distance and are more vulnerable to both amplitude fluctuations and reverberations, but concluded that reverberation has a more severe effect than amplitude fluctuation, and that this means that frequencies above 8 kHz are not suitable for long distance communication.

**Figure 9:**
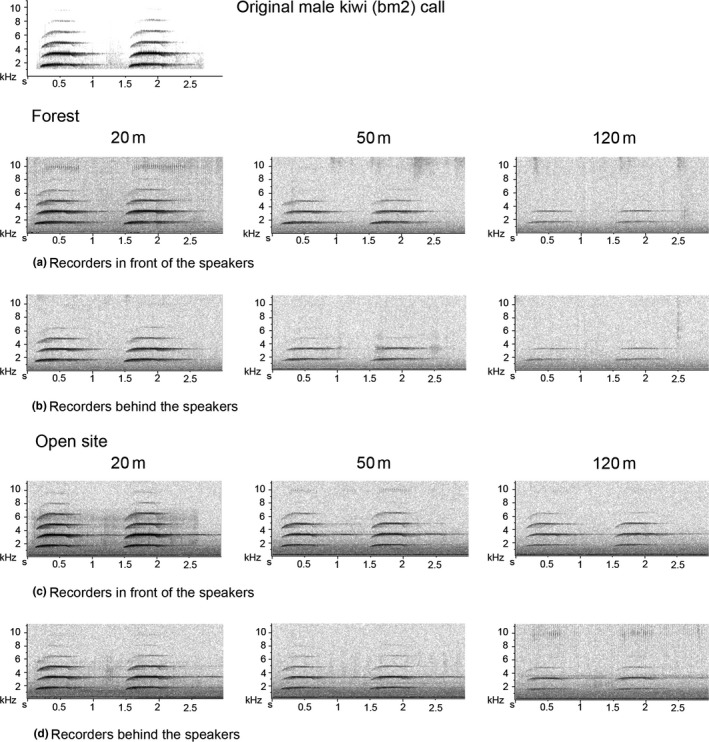
Recaptured male kiwi call (bm2) that was transmitted close to the ground in the forest and the open field at night (FNC and ONC, respectively)

Irregular amplitude fluctuations, mainly caused by atmospheric turbulence from the wind, are more severe in open fields than closed forest habitats and mask low frequencies. Reverberations are mainly generated by the scattering of sound from reflective surfaces such as tree trunks and foliage surfaces and are hence more relevant to forest habitats and mask high frequencies (Wiley & Richards, 1978). Spectrograms get blurry and tonal sounds with sharp start and end arrive at the receiver with progressive onset and long reverberations (Figure 9) because omnidirectional recorders (automated recorders) pick up the signals that are scattered and reflected by trees and other obstacles (Agranat, 2009; Wiley & Richards, 1978).

Our findings of sound attenuation in relation to distance and frequency are in partial agreement with Richards and Wiley (1980) and Wiley and Richards (1978). We observed that in the open field at moderate distance, low frequencies suffered more attenuation. In contrast, in the forest, higher frequencies were attenuated more while the lower frequencies travelled further. It is clear from Figure 9 that the first harmonic (just below 2 kHz) was attenuated more in the open site than in the forest. In the open field, even the furthest recorder (120 m) captured 3–4 harmonics, but in the forest, the furthest recorder only captured two harmonics.

The acoustic adaption hypothesis suggests that rapid amplitude modulations (high‐frequency trills) and low‐frequency amplitude modulations (whistles) are more appropriate for open and closed habitats, respectively (Brown & Handford, 2000). We found this to be largely true for ground birds. Morton (1975) suggested that narrow‐frequency tone‐like sounds are more suitable for forest birds living close to the ground, which is true particularly for male kiwi and weka; when these calls were captured at relatively large distances (>100 m), only the fundamental frequency component and the first harmonic (<2.5 kHz) remained (see Figure 9).

Ken et al. (1977) reported that habitat had limited effect on sound attenuation. However, our results, using real bird sounds broadcast at two heights and two differing habitats, suggest that the habitat has a larger effect on birdsong capture than does transmission height (Table 4, Figure 3, Table S4.2, and Table S4.4). The selected bird sounds were less attenuated in the forest than in the open field; the only exception was the very low‐frequency booms that transmitted almost equally in both habitats.

Previous studies have shown that ground attenuation is highest at frequencies below 1 kHz in open space and also depends on the transmission height and the distance to the recorder in relation to the wavelength (Aylor, 1972; Ingard, 1953; Ken et al., 1977; Linskens et al., 1976; Marten & Marler, 1977). Note that we used a fixed height for the recorder (1.5 m) and changed the transmission height, but most of the other researchers (Ingard, 1953; Maciej, Fischer, & Hammerschmidt, 2011; Marten & Marler, 1977; Richards & Wiley, 1980) located the source and the receiver at the same height. By broadcasting white noise and pure tones (0.3–11 kHz) in temperate and tropical habitats, Marten and Marler (1977) found that transmission height had the greatest effect on sound attenuation followed by frequency at the source: There was a higher attenuation for higher frequencies. Therefore, in most cases, lower frequencies were transmitted better. However, Marten and Marten and Marler (1977) also found that very low‐frequency sounds produced close to the ground attenuated more, particularly in open areas. They suggested that this is due to the interaction with the ground surface and micrometeorological events at the air‐ground interface such as air turbulence and temperature gradients (these effects are higher in open habitat than forest). Somewhat different to the findings of Marten and Marler (1977) and Maciej et al. (2011) experienced a stronger attenuation of primate vocalization in the forest compared to the open habitat. They mentioned that the signal attenuation was strongest in dense forest with low transmission height (0.5 m) and lowest in the open field with high transmission height (2 m).

In this study, we found higher attenuation when the speaker was close to the ground, not only in the open site, but also in the forest. Female kiwis, weka, and morepork sounds were more attenuated when the bird was close to the ground (0.25 m) than at 3 m height (Figure 3), the attenuation in the forest was always lower than in the open field (Figure S4.2), and the difference was higher during the night (Figure 2b). The calling posture of at least two kiwi species, brown and little spotted (Digby, 2013), could be their natural adjustment to this: Both adopt a unique calling posture, extending the neck and pointing the bill upwards so that their sounds can probably avoid some ground attenuation. The North Island robin is a ground forager and hihi use the ground for foraging and copulating, and hence, both species mainly call under the canopy. Our experiments demonstrated that North Island robin and hihi sounds were better transmitted close to the ground, in line with their behavior. While moreporks naturally roost on a reasonably high tree branch, this phenomenon may not have major consequences to their communication.

The interaction between the transmission height and the site was also significant for some bird sounds: kākāpō chinging, hihi, and North Island robin (Figure S4.3). In contrast to Marten and Marler (1977), our experiments have shown that in the forest, the impact of transmission height was higher than in the open site. The chinging sound of flightless kākāpō was best transmitted at ground level in the open site while transmitted equally at two transmission heights in the forest. However, it is worth noting that even though kākāpō cannot fly, they are great climbers, and also stretch out their necks when calling, as kiwi do.

Considering directionality of the sound, Richards and Wiley (1980) suggested that in scattering environments (e.g., forest), optimal directionality of sound production and reception is advantageous, but this does not seem to have been studied before. We found that there was a significant effect of the direction of the bird call relevant to the recorder, on birdsong acquisition. Interestingly, the recorders behind the bird (Dir3) recorded the bird sounds better than the recorders on the two other directions. We attribute this effect to the light wind, blowing from Dir1.

## 5 CONCLUSIONS

Automated recognition of birdsong is challenging in the presence of faint calls, which are a major cause of false positives (Cragg, Burger, & Piatt, 2015; Digby, Towsey, Bell, & Teal, 2013; Potamitis et al., 2014). The purpose of this manuscript was to explore how different bird calls attenuate under different environmental conditions primarily to facilitate the development of protocols for birdsong acquisition using automated recorders. The experiments confirmed that the forest consistently caused lower attenuation compared to an open site. This result encourages the use of autonomous recorders in the forest. Song acquisition of nocturnal birds was best during the night in line with natural selection of species song. However, the transmission height was an issue for some flightless birds resulting in more attenuation when the speaker was close to the ground, particularly for female kiwi and weka. Nevertheless, we suggest that birds compensate for this disadvantage by adjusting their calling posture. Morepork vocalizations were better captured when the bird was above the ground under this recorder positioning (at eye level), confirming that the current practice of morepork data collection is suitable.

The effect of wind was severe in the open site and was minimal under the canopy in the forest. Therefore, the field recordings collected in forested habitats are less susceptible to direct effect from the wind, but indirect effects such as boosting the rustling noise of leaves can still affect the recordings. According to the results of this study, the main advantage in the forested habitats is that bird songs are not guided by the wind, which means the recorder positioning is essentially comfortable compared to an open field. However, the directionality of bird calls still plays a role in field recordings.

The study confirmed that higher frequencies attenuate more with distance, especially in the forest. Frequencies above 8 kHz were largely attenuated even at a moderate distance. Due to this frequency selective attenuation of bird sounds, selecting a large sampling frequency, particularly when using autonomous recorders to capture birdsong, may increase the volume of data unnecessarily. For example, when recording kiwi and morepork, we suggest that 16 kHz is more suitable and we still obtain almost the same data as at 48 kHz. Recording at the lower frequency results in significant storage savings, and it is also a way to avoid the species that are beyond the frequency of the target species.

The next steps following this study are to include models of birdsong attenuation with distance into distance sampling models to enable methods that perform birdsong recognition and classification to reliably estimate the number of birds present in an area based on acoustic recordings, factoring in the weather effects. For this to be reliable, it will be necessary to investigate the effects of topography and other confounding factors on birdsong transmission in different habitats. In this way, we hope to enable the aims of ecoacoustics to be achieved, allowing environmental sound to act as a reliable proxy for ecological complexity.

## CONFLICT OF INTEREST

None declared.

## AUTHOR CONTRIBUTIONS

NP, IC, and SM conceived and designed the research; NP conducted the experiments; NP, IC, and SM designed and conducted the analyses and interpretation; NP, IC, and SM wrote the manuscript.

## DATA ACCESSIBILITY

Data available from http://avianz.massey.ac.nz/.
