# Supplementary figures and images for: The impact of environmental factors in birdsong acquisition using automated recorders
Source: Ecol Evol. 2018 Apr 24;8(10):5016–33. doi: 10.1002/ece3.3889 (PMC5980359; doi:10.1002/ece3.3889)

EM Means

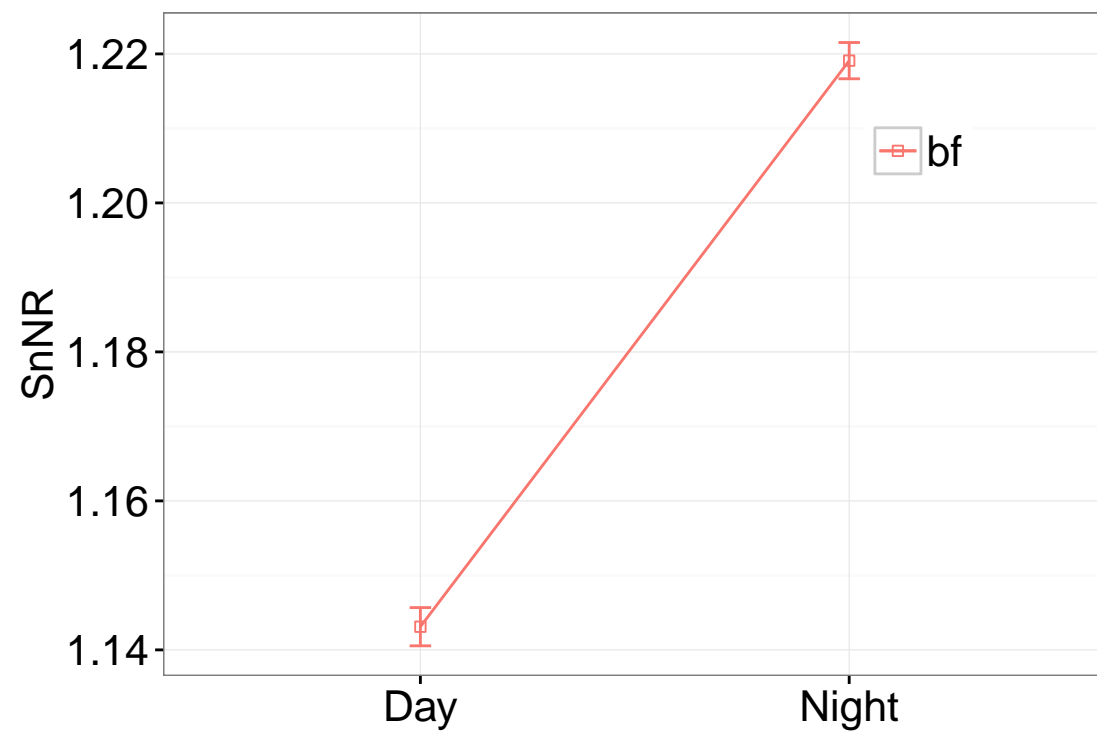

EM Means

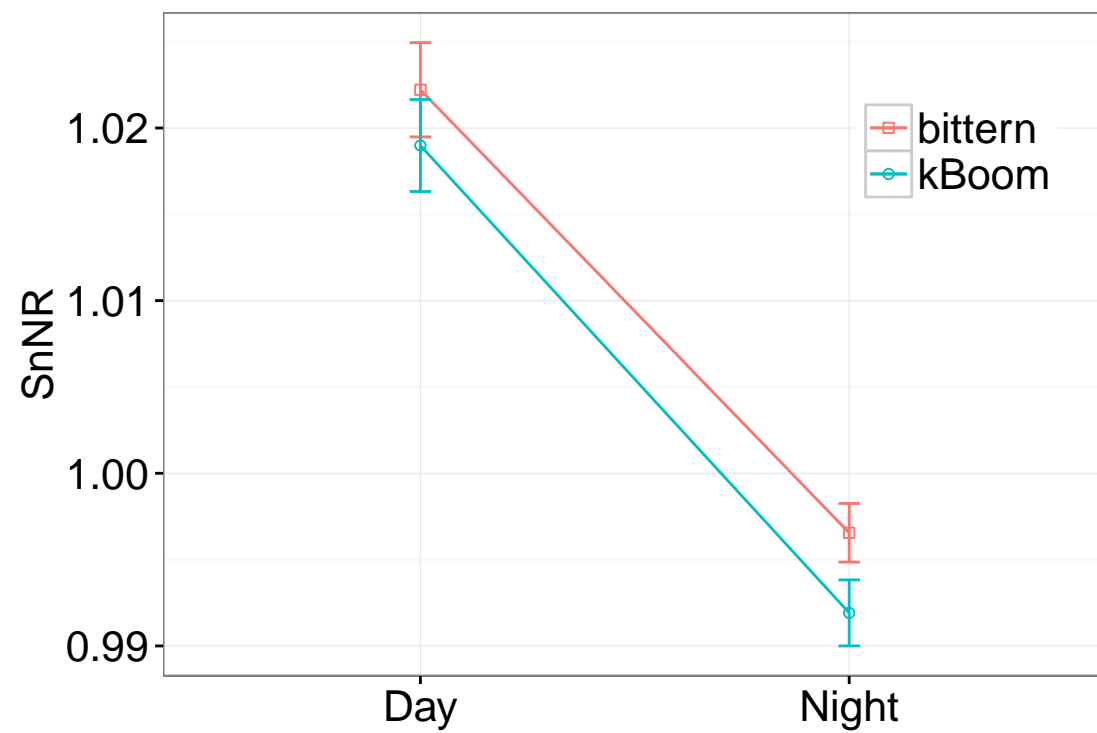

EM Means

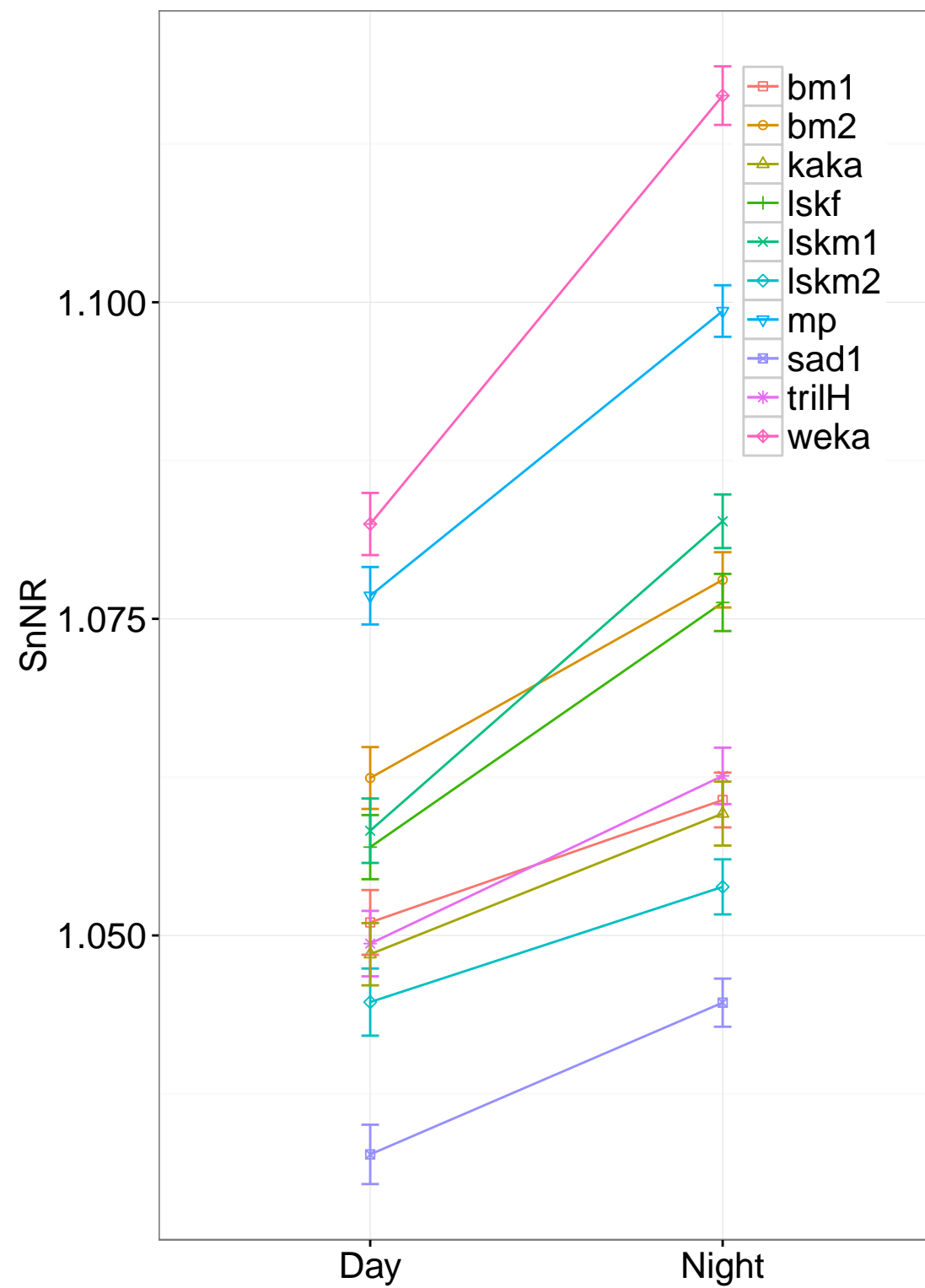

Supplement: Supplementary file 1 [file ECE3-8-5016-s001.pdf]

## EM Means

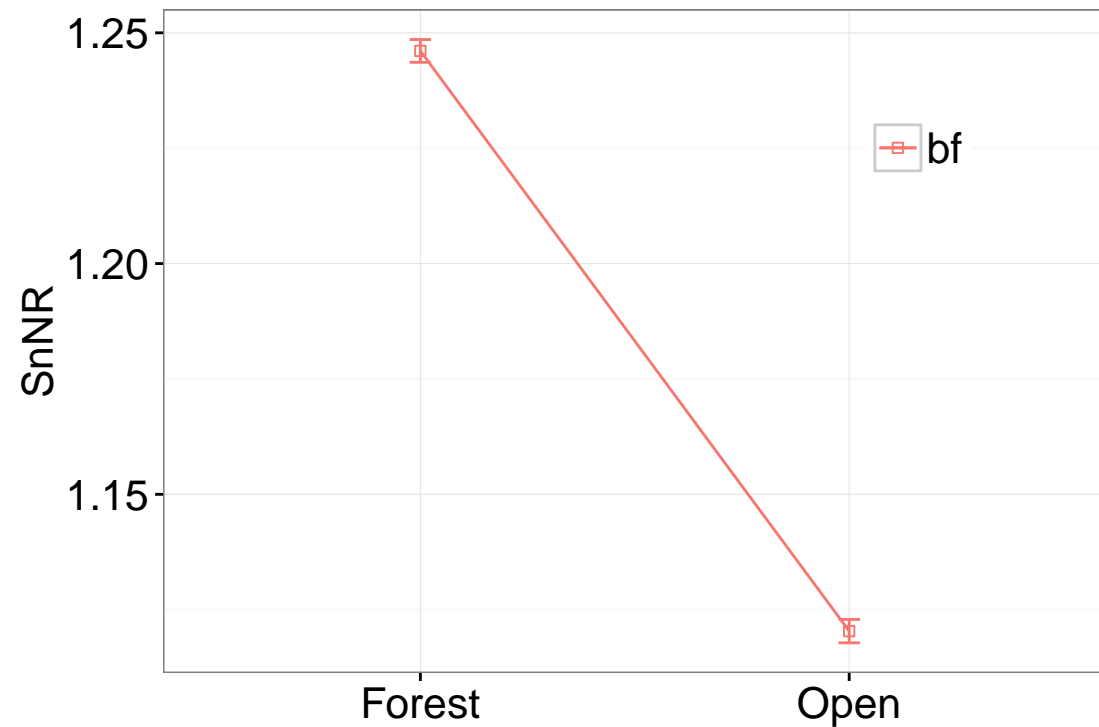

## EM Means

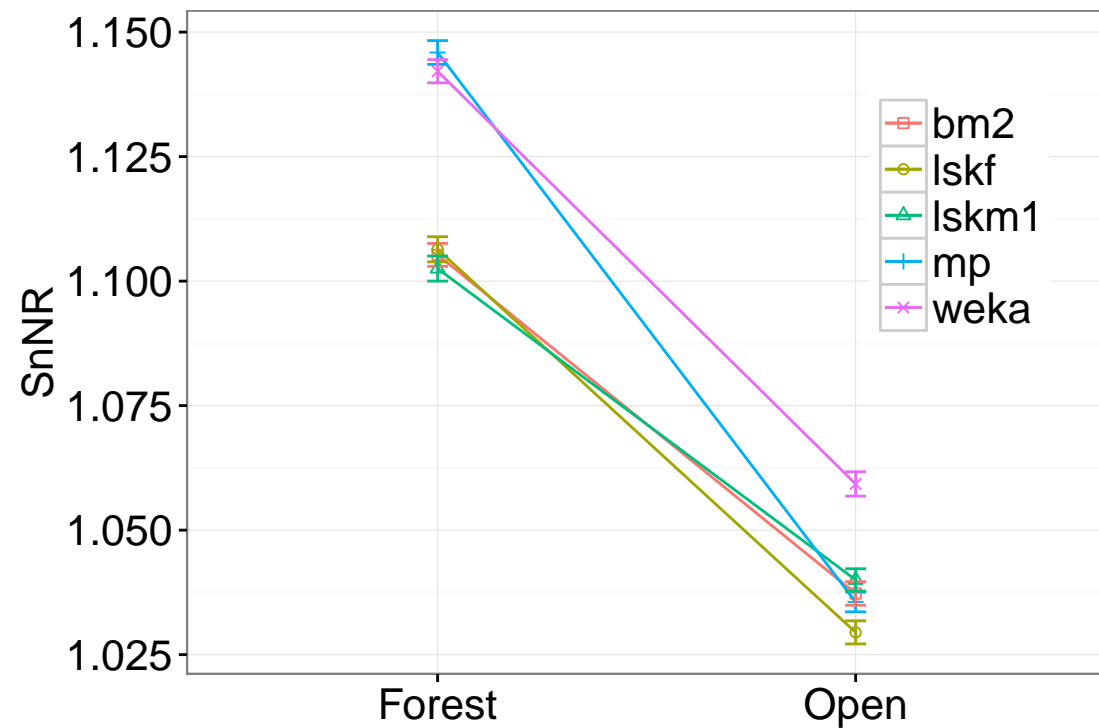

## EM Means

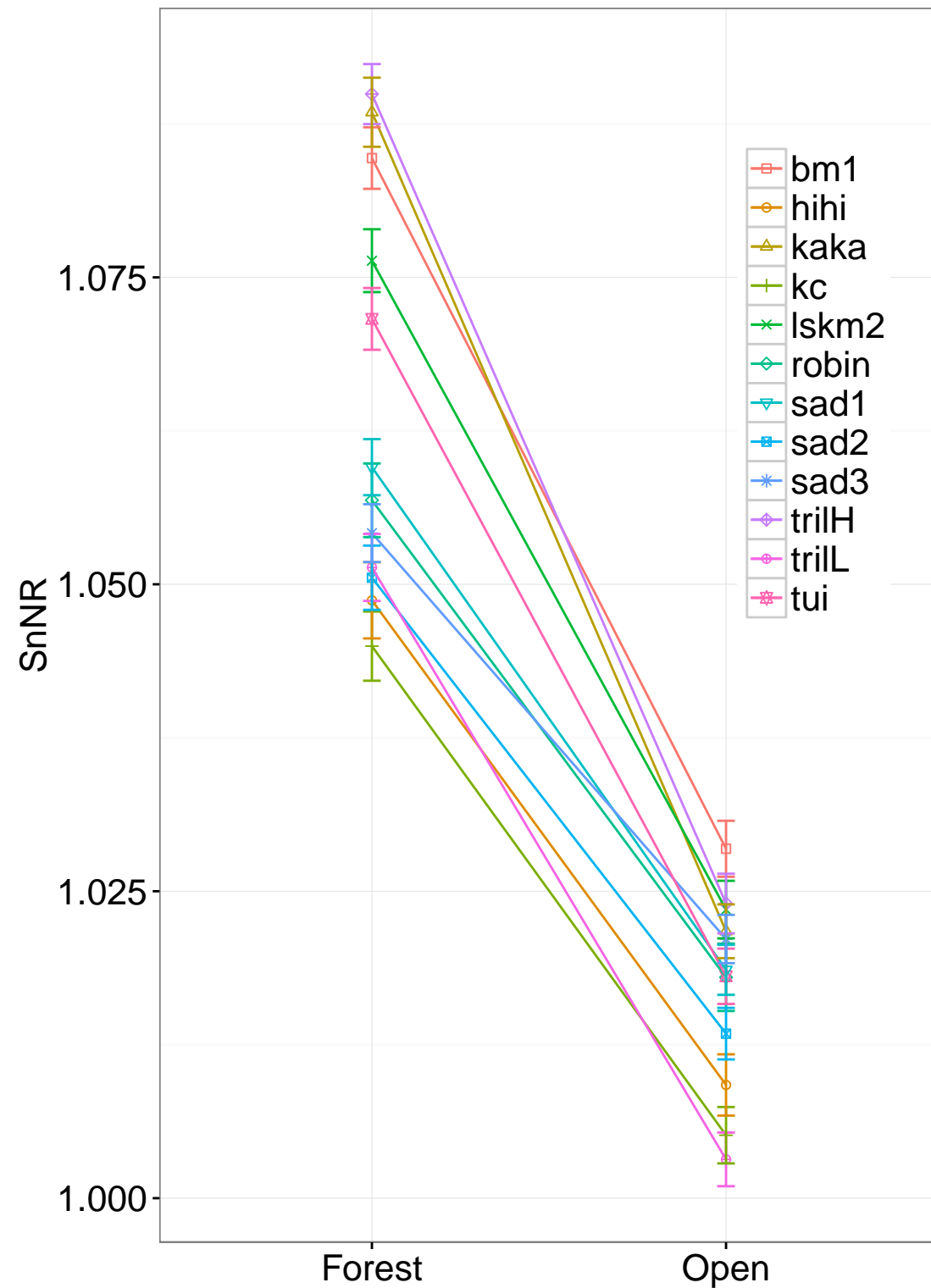

Supplement: Supplementary file 2 [file ECE3-8-5016-s002.pdf]

EMM – bf

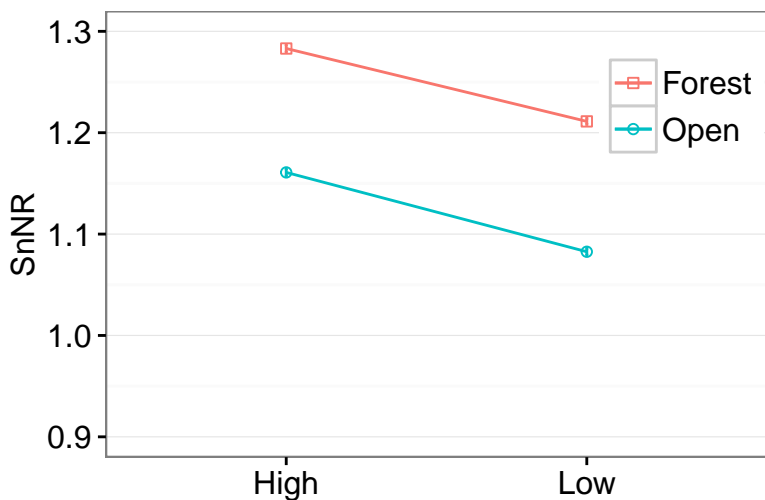

EMM – bm2

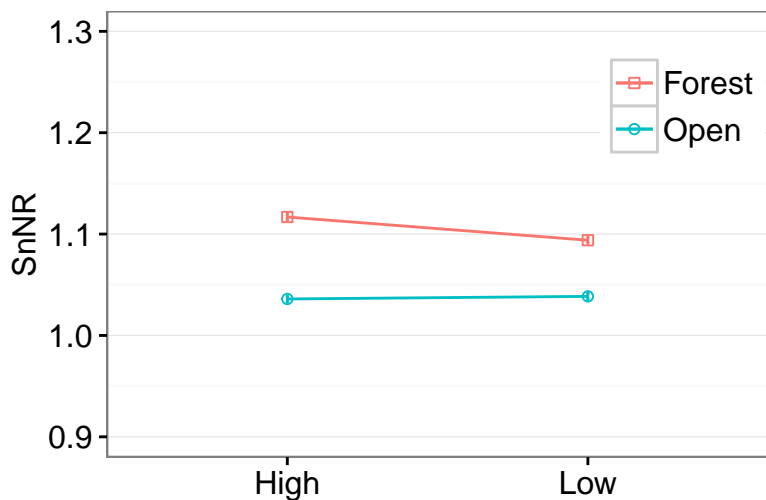

EMM – lskf

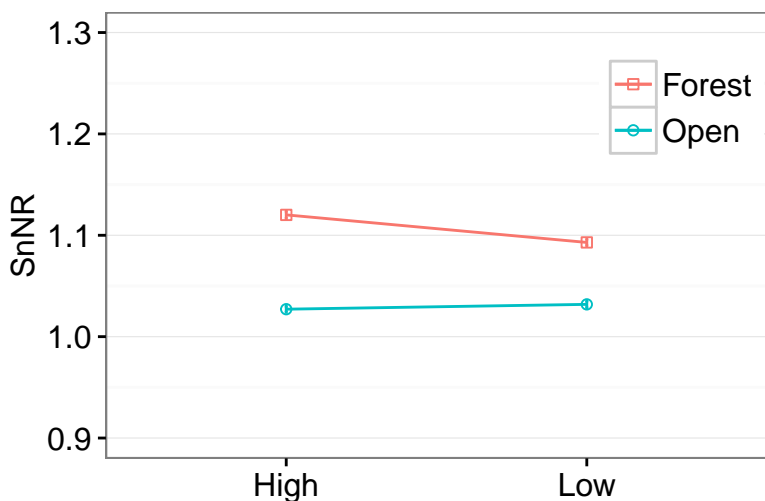

EMM – mp

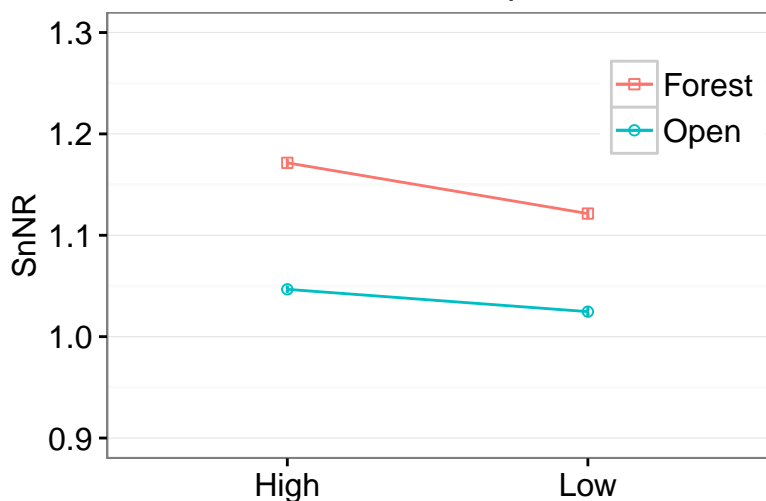

EMM – trilH

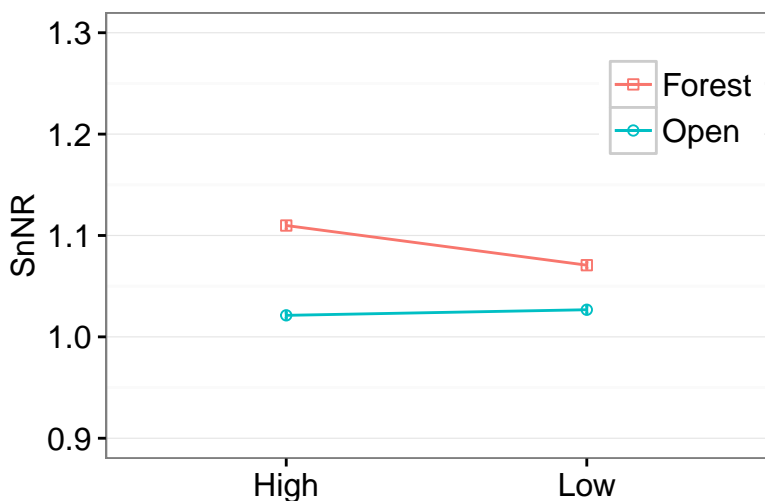

EMM – kc

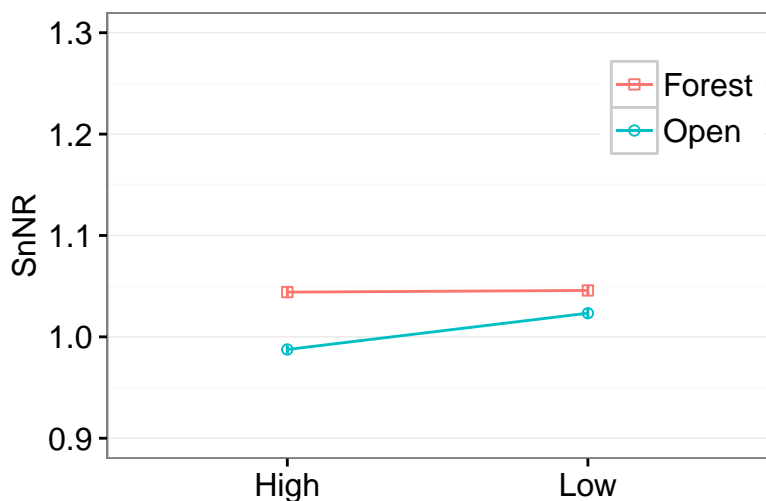

EMM – hihi

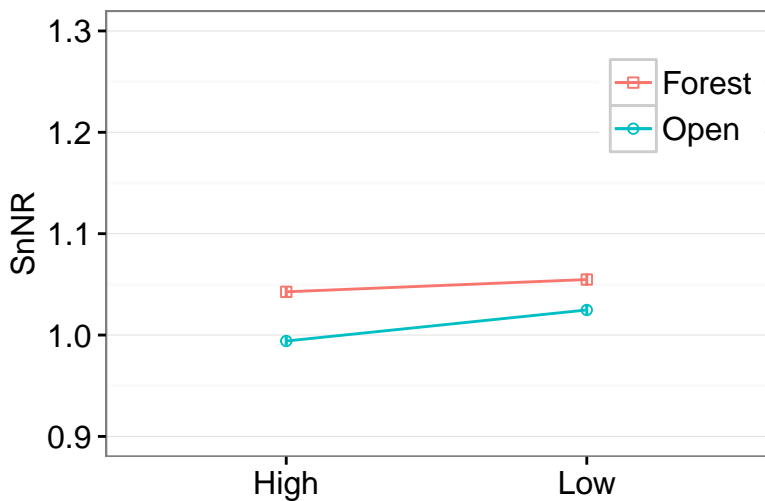

EMM – robin

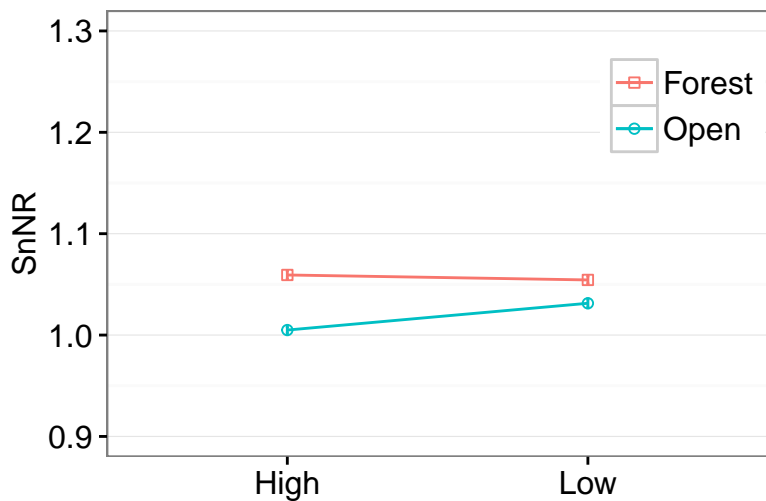

Supplement: Supplementary file 3 [file ECE3-8-5016-s003.pdf]

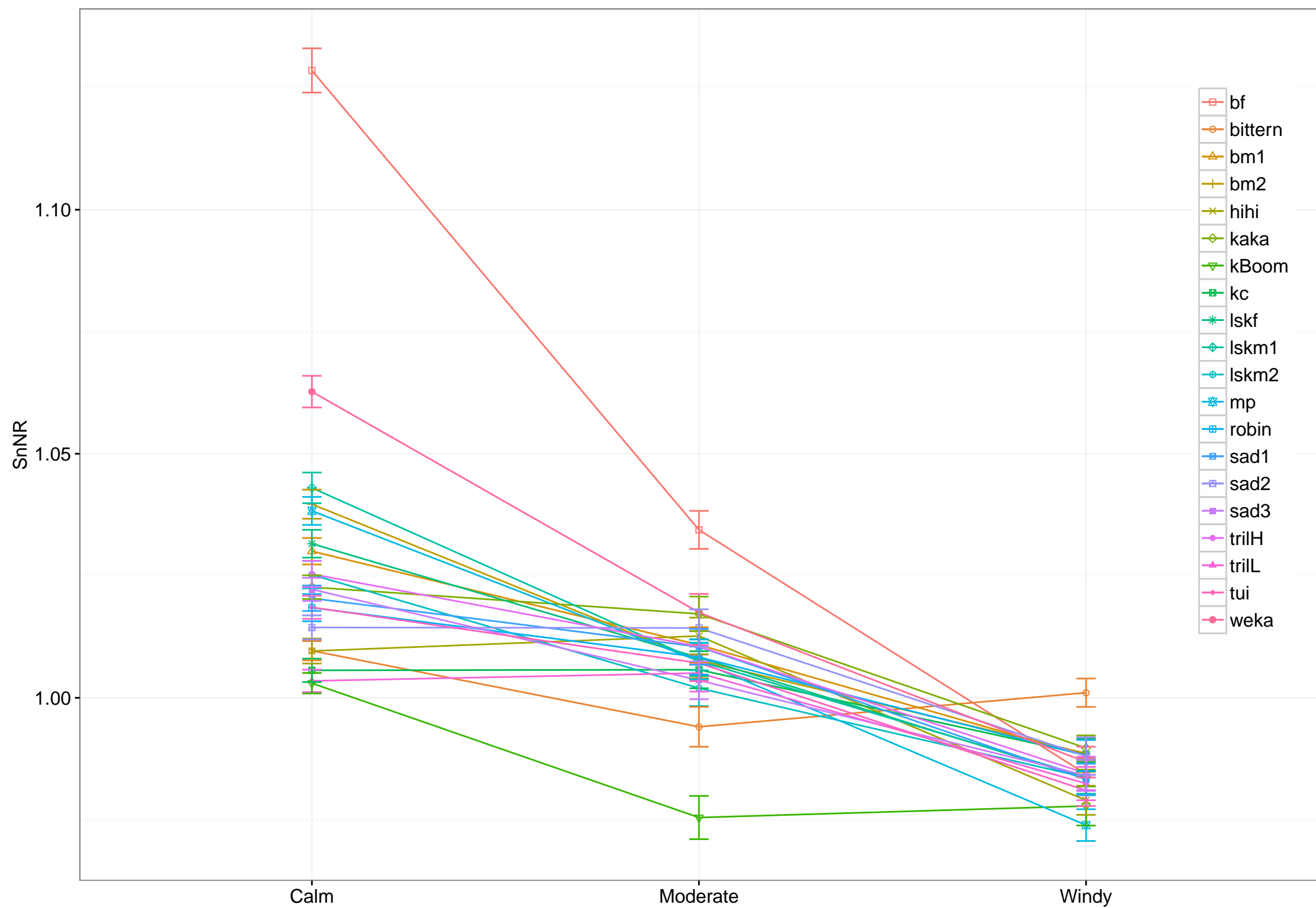

Supplement: Supplementary file 4 [file ECE3-8-5016-s004.pdf]
