# Supplementary material for: The impact of environmental factors in birdsong acquisition using automated recorders
Source: Ecol Evol. 2018 Apr 24;8(10):5016–33. doi: 10.1002/ece3.3889 (PMC5980359; doi:10.1002/ece3.3889)
Supplement: Supplementary file 5 [file ECE3-8-5016-s005.pdf]

The Impact of Environmental Factors in Birdsong Acquisition using Automated Recorders

S4 Generalised Linear Models for each analysis

Table 1: Model effects. Dependent variable is SnNR.

Call example	Model effect
bf (brown kiwi female)	Tests of Model Effects - bf (see below)
bm1 (brown kiwi male)	Tests of Model Effects - bm1 (see below)
bm2 (brown kiwi male)	Tests of Model Effects - bm2 (see below)

Tests of Model Effects - bf

Source	Type III Wald Chi-Square	df	Sig.
(Intercept)	435926.413	1	.000
DayNight	458.940	1	.000
OpenForest	1227.892	1	.000
Height	455.003	1	.000
RDirection	62.909	3	.000
Distance	5336.644	4	.000
DayNight * OpenForest	12.557	1	.000
DayNight * Height	37.118	1	.000
DayNight * RDirection	25.307	3	.000
DayNight * Distance	35.076	4	.000
OpenForest * Height	10.088	1	.001
OpenForest * RDirection	31.136	3	.000
OpenForest * Distance	618.507	4	.000
Height * Distance	23.324	4	.000
RDirection * Distance	94.525	12	.000

Tests of Model Effects - bm1

Source	Type III Wald Chi-Square	df	Sig.
(Intercept)	391762.615	1	.000
DayNight	8.414	1	.004
OpenForest	276.372	1	.000
RDirection	39.304	3	.000
Distance	1545.810	4	.000
DayNight * RDirection	30.015	3	.000
DayNight * Distance	105.438	4	.000
OpenForest * Distance	287.972	4	.000
RDirection * Distance	73.657	12	.000

Tests of Model Effects - bm2

Source	Type III Wald Chi-Square	df	Sig.
(Intercept)	420063.931	1	.000
DayNight	23.049	1	.000
OpenForest	419.823	1	.000
Height	8.193	1	.004
RDirection	34.103	3	.000
Distance	2292.386	4	.000
DayNight * OpenForest	11.669	1	.001
DayNight * Height	22.944	1	.000
DayNight * RDirection	37.997	3	.000
DayNight * Distance	131.733	4	.000
OpenForest * Height	13.147	1	.000
OpenForest * Distance	418.248	4	.000
Height * Distance	27.620	4	.000
RDirection * Distance	76.511	12	.000

lskf
(little spotted kiwi female)

Tests of Model Effects - lskf

Source	Type III		
	Wald Chi-Square	df	Sig.
(Intercept)	388443.209	1	.000
DayNight	32.719	1	.000
OpenForest	505.603	1	.000
Height	8.854	1	.003
RDirection	20.074	3	.000
Distance	1841.466	4	.000
DayNight * Height	45.319	1	.000
DayNight * RDirection	27.660	3	.000
DayNight * Distance	84.729	4	.000
OpenForest * Height	19.749	1	.000
OpenForest * Distance	276.773	4	.000
Height * Distance	47.699	4	.000
RDirection * Distance	58.597	12	.000

lskm1
(little spotted kiwi male)

Tests of Model Effects - lskm1

Source	Type III		
	Wald Chi-Square	df	Sig.
(Intercept)	410010.457	1	.000
DayNight	54.633	1	.000
OpenForest	346.962	1	.000
RDirection	38.052	3	.000
Distance	2405.542	4	.000
DayNight * OpenForest	11.260	1	.001
DayNight * RDirection	58.313	3	.000
DayNight * Distance	143.316	4	.000
OpenForest * Distance	286.642	4	.000
RDirection * Distance	65.743	12	.000

lskm2
(little spotted kiwi male)

Tests of Model Effects - lskm2

Source	Type III		
	Wald Chi-Square	df	Sig.
(Intercept)	368738.967	1	.000
DayNight	7.099	1	.008
OpenForest	232.039	1	.000
RDirection	21.583	3	.000
Distance	1467.397	4	.000
DayNight * OpenForest	7.374	1	.007
DayNight * RDirection	33.928	3	.000
DayNight * Distance	120.392	4	.000
OpenForest * Distance	219.223	4	.000
RDirection * Distance	73.096	12	.000

mp
(more-pork sound
of morepork)

Tests of Model Effects - mp

Source	Type III		
	Wald Chi-Square	df	Sig.
(Intercept)	502517.002	1	.000
DayNight	54.713	1	.000
OpenForest	1295.983	1	.000
Height	131.693	1	.000
RDirection	35.373	3	.000
Distance	2681.364	4	.000
DayNight * Height	91.434	1	.000
DayNight * RDirection	37.279	3	.000
DayNight * Distance	110.050	4	.000
OpenForest * Height	12.285	1	.000
OpenForest * RDirection	21.292	3	.000
OpenForest * Distance	479.029	4	.000
Height * RDirection	13.267	3	.004
Height * Distance	154.768	4	.000
RDirection * Distance	78.997	12	.000

trilH
(trill sound
of morepork)

Tests of Model Effects - trilH

Source	Type III		
	Wald Chi-Square	df	Sig.
(Intercept)	376264.971	1	.000
DayNight	15.220	1	.000
OpenForest	361.261	1	.000
Height	20.200	1	.000
Distance	1520.474	4	.000
DayNight * Height	24.568	1	.000
DayNight * Distance	99.755	4	.000
OpenForest * Height	36.902	1	.000
OpenForest * Distance	324.236	4	.000
Height * Distance	25.857	4	.000

trilL(trill sound
of morepork)

Tests of Model Effects - trilL

Source	Type III		
	Wald Chi-Square	df	Sig.
(Intercept)	351003.071	1	.000
OpenForest	193.304	1	.000
Distance	558.507	4	.000
OpenForest * Distance	146.340	4	.000

bittern

Tests of Model Effects - bittern

Source	Type III		
	Wald Chi-Square	df	Sig.
(Intercept)	402875.183	1	.000
DayNight	65.031	1	.000
RDirection	20.533	3	.000
Distance	216.849	4	.000
DayNight * Distance	35.465	4	.000
RDirection * Distance	47.662	12	.000

kBoom
(kākāpō boom)

Tests of Model Effects - kBoom

Source	Type III		
	Wald Chi-Square	df	Sig.
(Intercept)	381696.379	1	.000
DayNight	69.276	1	.000
RDirection	17.212	3	.001
BDirection	20.533	3	.000
Distance	142.734	4	.000
DayNight * BDirection	68.076	3	.000
DayNight * Distance	27.683	4	.000
RDirection * Distance	35.222	12	.000

kc
(kākāpō chinging)

Tests of Model Effects - kc

Source	Type III		
	Wald Chi-Square	df	Sig.
(Intercept)	322419.016	1	.000
OpenForest	121.512	1	.000
Height	29.193	1	.000
Distance	473.442	4	.000
OpenForest * Height	24.233	1	.000
OpenForest * Distance	73.503	4	.000
Height * Distance	16.704	4	.002

Tests of Model Effects - weka

Source	Type III Wald Chi-Square	df	Sig.
(Intercept)	418456.680	1	.000
DayNight	101.130	1	.000
OpenForest	587.728	1	.000
Height	69.573	1	.000
RDirection	65.460	3	.000
Distance	3064.944	4	.000
DayNight * OpenForest	22.086	1	.000
DayNight * Height	10.504	1	.001
DayNight * RDirection	21.549	3	.000
DayNight * Distance	119.356	4	.000
OpenForest * RDirection	18.605	3	.000
OpenForest * Distance	548.977	4	.000
Height * Distance	16.513	4	.002
RDirection * Distance	82.578	12	.000

weka

Tests of Model Effects - kaka

Source	Type III Wald Chi-Square	df	Sig.
(Intercept)	358669.103	1	.000
DayNight	9.937	1	.002
OpenForest	358.353	1	.000
Distance	1063.572	4	.000
DayNight * Distance	87.049	4	.000
OpenForest * Distance	183.242	4	.000

kaka

Tests of Model Effects - hihi

Source	Type III Wald Chi-Square	df	Sig.
(Intercept)	270657.482	1	.000
OpenForest	99.577	1	.000
Height	30.223	1	.000
Distance	374.643	4	.000
OpenForest * Height	6.496	1	.011
OpenForest * Distance	54.688	4	.000

hihi

Tests of Model Effects - robin

Source	Type III Wald Chi-Square	df	Sig.
(Intercept)	261386.084	1	.000
OpenForest	91.663	1	.000
Height	7.682	1	.006
Distance	645.318	4	.000
OpenForest * Height	15.379	1	.000
OpenForest * Distance	118.373	4	.000

robin

Tests of Model Effects - tui

Source	Type III Wald Chi-Square	df	Sig.
(Intercept)	385436.967	1	.000
OpenForest	252.596	1	.000
RDirection	19.807	3	.000
Distance	923.333	4	.000
OpenForest * RDirection	19.526	3	.000
OpenForest * Distance	254.815	4	.000
RDirection * Distance	57.663	12	.000

tui

Tests of Model Effects - sad1

Source	Type III Wald Chi-Square	df	Sig.
(Intercept)	468173.099	1	.000
DayNight	15.806	1	.000
OpenForest	178.714	1	.000
RDirection	20.618	3	.000
BDirection	27.320	3	.000
Distance	1387.403	4	.000
DayNight * OpenForest	6.450	1	.011
DayNight * RDirection	43.623	3	.000
DayNight * BDirection	21.213	3	.000
DayNight * Distance	123.319	4	.000
OpenForest * BDirection	25.179	3	.000
OpenForest * Distance	240.741	4	.000
RDirection * BDirection	155.886	9	.000
RDirection * Distance	87.001	12	.000

sad1 (saddleback)

Tests of Model Effects - sad2

Source	Type III Wald Chi-Square	df	Sig.
(Intercept)	386868.562	1	.000
OpenForest	122.999	1	.000
RDirection	29.193	3	.000
BDirection	23.945	3	.000
Distance	916.648	4	.000
OpenForest * Distance	176.392	4	.000
RDirection * BDirection	179.329	9	.000
RDirection * Distance	70.407	12	.000

sad2 (saddleback)

Tests of Model Effects - sad3

Source	Type III Wald Chi-Square	df	Sig.
(Intercept)	463032.976	1	.000
OpenForest	116.773	1	.000
RDirection	47.418	3	.000
BDirection	34.751	3	.000
Distance	1328.026	4	.000
OpenForest * RDirection	12.942	3	.005
OpenForest * BDirection	27.592	3	.000
OpenForest * Distance	301.649	4	.000
RDirection * BDirection	126.869	9	.000
RDirection * Distance	71.048	12	.000

sad3 (saddleback)

Table 2: EMM – Open vs Forest – overall test results – significant effects after sequential Sidak correction on $\alpha = 0.01$.

Call example	Wald Chi-Square	df	Significance
bf	1262.722	1	0.000
bm1	275.152	1	0.000
bm2	424.367	1	0.000
lskf	504.704	1	0.000
lskm1	344.597	1	0.000
lskm2	231.155	1	0.000
mp	1274.189	1	0.000
trilH	363.747	1	0.000
trilL	190.194	1	0.000
bittern	Experiment site was not in the model		
kBoom	Experiment site was not in the model		
kc	119.971	1	0.000
weka	598.767	1	0.000
kaka	350.096	1	0.000
hihi	98.226	1	0.000
robin	91.299	1	0.000
tui	251.081	1	0.000
sad1	177.674	1	0.000
sad2	121.402	1	0.000
sad3	115.682	1	0.000

Table 3: EMM – Day vs Night – overall test results – significant effects after sequential Sidak correction on $\alpha = 0.01$.

**Call example**	**Wald Chi-Square**	**df**	**Significance**
bf	465.943	1	0.000
bm1	8.441	1	0.004
bm2	23.136	1	0.000
lskf	32.878	1	0.000
lskm1	55.147	1	0.000
lskm2	7.125	1	0.008
mp	55.008	1	0.000
trilH	15.282	1	0.000
trilL	Time of day was not in the model		
bittern	63.653	1	0.000
kBoom	68.178	1	0.000
kc	Time of day was not in the model		
weka	101.695	1	0.000
kaka	9.934	1	0.002
hihi	Time of day was not in the model		
robin	Time of day was not in the model		
tui	Time of day was not in the model		
sad1	15.886	1	0.000
sad2	Time of day was not in the model		
sad3	Time of day was not in the model		

Table 4: EMM – Low vs High transmission height – overall test results – significant effects after sequential Sidak correction on $\alpha = 0.01$.

Call example	Wald Chi-Square	df	Significance
bf	450.625	1	0.000
bm1	Transmission height was not in the model		
bm2	8.207	1	0.004
lskf	8.861	1	0.003
lskm1	Transmission height was not in the model		
lskm2	Transmission height was not in the model		
mp	133.562	1	0.000
trilH	20.228	1	0.000
trilL	Transmission height was not in the model		
bittern	Transmission height was not in the model		
kBoom	Transmission height was not in the model		
kc	29.041	1	0.000
weka	69.261	1	0.000
kaka	Transmission height was not in the model		
hihi	29.929	1	0.000
robin	7.673	1	0.006
tui	Transmission height was not in the model		
sad1	Transmission height was not in the model		
sad2	Transmission height was not in the model		
sad3	Transmission height was not in the model		

Table 5: EMM – Distance (20m, 25m, 50m, 100m, 120m) – individual test results – significant effects after sequential Sidak correction on $\alpha = 0.01$.

Call example	Individual Test Results	Contrast Estimate	Std. Error	Wald Chi-Square	df	Significance
bf	25m vs. 20m	-0.055	0.007	71.325	1	0.000
	50m vs. 25m	-0.159	0.005	896.085	1	0.000
	100m vs. 50m	-0.225	0.004	2522.045	1	0.000
	120m vs. 100m	-0.183	0.004	2300.891	1	0.000
bm1	25m vs. 20m	-0.045	0.006	55.449	1	0.000
	50m vs. 25m	-0.109	0.005	547.44	1	0.000
	100m vs. 50m	-0.111	0.004	746.607	1	0.000
	120m vs. 100m	-0.077	0.004	353.357	1	0.000
bm2	25m vs. 20m	-0.042	0.006	46.193	1	0.000
	50m vs. 25m	-0.122	0.005	643.257	1	0.000
	100m vs. 50m	-0.137	0.004	1136.891	1	0.000
	120m vs. 100m	-0.098	0.004	718.888	1	0.000
lskf	25m vs. 20m	-0.035	0.006	32.388	1	0.000
	50m vs. 25m	-0.114	0.005	573.856	1	0.000
	100m vs. 50m	-0.127	0.004	930.363	1	0.000
	120m vs. 100m	-0.093	0.004	514.513	1	0.000
lskm1	25m vs. 20m	-0.039	0.006	46.925	1	0.000
	50m vs. 25m	-0.127	0.005	725.696	1	0.000
	100m vs. 50m	-0.136	0.004	1097.447	1	0.000
	120m vs. 100m	-0.099	0.004	616.232	1	0.000
lskm2	25m vs. 20m	-0.036	0.006	35.918	1	0.000
	50m vs. 25m	-0.107	0.005	450.484	1	0.000
	100m vs. 50m	-0.11	0.004	683.962	1	0.000
	120m vs. 100m	-0.076	0.004	370.313	1	0.000
mp	25m vs. 20m	-0.036	0.005	53.744	1	0.000
	50m vs. 25m	-0.109	0.004	652.662	1	0.000
	100m vs. 50m	-0.139	0.004	1230.545	1	0.000
	120m vs. 100m	-0.104	0.004	827.859	1	0.000
trilH	25m vs. 20m	-0.043	0.006	44.388	1	0.000
	50m vs. 25m	-0.108	0.005	480.68	1	0.000
	100m vs. 50m	-0.117	0.004	789.155	1	0.000
	120m vs. 100m	-0.08	0.004	409.618	1	0.000
trilL	25m vs. 20m	-0.034	0.006	30.555	1	0.000
	50m vs. 25m	-0.068	0.005	196.043	1	0.000
	100m vs. 50m	-0.071	0.004	283.129	1	0.000
	120m vs. 100m	-0.043	0.004	106.882	1	0.000
bittern	25m vs. 20m	-0.019	0.005	14.924	1	0.000
	50m vs. 25m	-0.033	0.005	49.011	1	0.000
	100m vs. 50m	-0.033	0.004	59.773	1	0.000
	120m vs. 100m	-0.031	0.004	73.72	1	0.000
kBoom	25m vs. 20m	-0.015	0.005	10.358	1	0.001
	50m vs. 25m	-0.021	0.005	19.516	1	0.000
	100m vs. 50m	-0.03	0.005	44.455	1	0.000
	120m vs. 100m	-0.027	0.004	51.843	1	0.000

Call example	Individual Test Results	Contrast Estimate	Std. Error	Wald Chi-Square	df	Significance
kc	25m vs. 20m	-0.029	0.006	21.804	1	0.000
	50m vs. 25m	-0.068	0.005	163.922	1	0.000
	100m vs. 50m	-0.062	0.004	202.343	1	0.000
	120m vs. 100m	-0.04	0.004	90.54	1	0.000
weka	25m vs. 20m	-0.048	0.006	56.396	1	0.000
	50m vs. 25m	-0.143	0.005	933.082	1	0.000
	100m vs. 50m	-0.165	0.004	1525.535	1	0.000
	120m vs. 100m	-0.126	0.004	1044.493	1	0.000
kaka	25m vs. 20m	-0.031	0.006	26.36	1	0.000
	50m vs. 25m	-0.091	0.005	363.913	1	0.000
	100m vs. 50m	-0.097	0.004	492.763	1	0.000
	120m vs. 100m	-0.067	0.004	242.535	1	0.000
hihi	25m vs. 20m	-0.028	0.007	15.518	1	0.000
	50m vs. 25m	-0.07	0.006	151.856	1	0.000
	100m vs. 50m	-0.062	0.005	163.31	1	0.000
	120m vs. 100m	-0.039	0.005	72.942	1	0.000
robin	25m vs. 20m	-0.032	0.008	15.717	1	0.000
	50m vs. 25m	-0.098	0.006	271.591	1	0.000
	100m vs. 50m	-0.083	0.005	308.766	1	0.000
	120m vs. 100m	-0.057	0.004	160.325	1	0.000
tui	25m vs. 20m	-0.03	0.006	23.896	1	0.000
	50m vs. 25m	-0.087	0.005	357.638	1	0.000
	100m vs. 50m	-0.089	0.004	452.081	1	0.000
	120m vs. 100m	-0.059	0.004	226.307	1	0.000
sad1	25m vs. 20m	-0.037	0.005	57.091	1	0.000
	50m vs. 25m	-0.099	0.004	520.542	1	0.000
	100m vs. 50m	-0.09	0.004	522.144	1	0.000
	120m vs. 100m	-0.057	0.004	255.396	1	0.000
sad2	25m vs. 20m	-0.032	0.006	33.856	1	0.000
	50m vs. 25m	-0.091	0.005	366.279	1	0.000
	100m vs. 50m	-0.079	0.004	353.125	1	0.000
	120m vs. 100m	-0.049	0.004	158.389	1	0.000
sad3	25m vs. 20m	-0.036	0.005	49.12	1	0.000
	50m vs. 25m	-0.098	0.004	569.688	1	0.000
	100m vs. 50m	-0.089	0.004	538.632	1	0.000
	120m vs. 100m	-0.059	0.004	242.381	1	0.000

Table 6: EMM – Distance (20m, 25m, 50m, 100m, 120m) – overall test results – significant effects after sequential Sidak correction on $\alpha = 0.01$.

Call example	Wald Chi-Square	df	Significance
bf	5120.568	4	0.000
bm1	1444.139	4	0.000
bm2	2115.337	4	0.000
lskf	1738.588	4	0.000
lskm1	2317.901	4	0.000
lskm2	1384.439	4	0.000
mp	2674.829	4	0.000
trilH	1405.971	4	0.000
trilL	530.703	4	0.000
bittern	213.906	4	0.000
kBoom	142.493	4	0.000
kc	452.054	4	0.000
weka	2848.775	4	0.000
kaka	1021.352	4	0.000
hihi	355.668	4	0.000
robin	587.483	4	0.000
tui	868.425	4	0.000
sad1	1342.053	4	0.000
sad2	878.114	4	0.000
sad3	1267.966	4	0.000

Table 7: EMM – Open/Forest*Day/Night interaction – overall test results – significant effects after sequential Sidak correction on $\alpha = 0.01$.

Call example	Wald Chi-Square	df	Significance
bf	1930.763	3	0.000
bm1	Open/Forest*Day/Night interaction was not in the model		
bm2	504.521	3	0.000
lskf	Open/Forest*Day/Night interaction was not in the model		
lskm1	438.395	3	0.000
lskm2	247.167	3	0.000
mp	Open/Forest*Day/Night interaction was not in the model		
trilH	Open/Forest*Day/Night interaction was not in the model		
trilL	Open/Forest*Day/Night interaction was not in the model		
bittern	Open/Forest*Day/Night interaction was not in the model		
kBoom	Open/Forest*Day/Night interaction was not in the model		
kc	Open/Forest*Day/Night interaction was not in the model		
weka	186.689	3	0.000
kaka	Open/Forest*Day/Night interaction was not in the model		
hihi	Open/Forest*Day/Night interaction was not in the model		
robin	Open/Forest*Day/Night interaction was not in the model		
tui	Open/Forest*Day/Night interaction was not in the model		
sad1	214.003	3	0.000
sad2	Open/Forest*Day/Night interaction was not in the model		
sad3	Open/Forest*Day/Night interaction was not in the model		

Table 8: EMM – Open/Forest*Low/High transmission interaction – overall test results – significant effects after sequential Sidak correction on $\alpha = 0.01$.

**Call example**	**Wald Chi-Square**	**df**	**Significance**
bf	1603.428	3	0.000
bm1	Open/Forest*Low/High was not in the model		
bm2	460.380	3	0.000
lskf	531.270	3	0.000
lskm1	Open/Forest*Low/High was not in the model		
lskm2	Open/Forest*Low/High was not in the model		
mp	1558.386	3	0.000
trilH	420.936	3	0.000
trilL	Open/Forest*Low/High was not in the model		
bittern	Open/Forest*Low/High was not in the model		
kBoom	Open/Forest*Low/High was not in the model		
kc	212.781	3	0.000
weka	Open/Forest*Low/High was not in the model		
kaka	Open/Forest*Low/High was not in the model		
hihi	159.311	3	0.000
robin	127.642	3	0.000
tui	Open/Forest*Low/High was not in the model		
sad1	Open/Forest*Low/High was not in the model		
sad2	Open/Forest*Low/High was not in the model		
sad3	Open/Forest*Low/High was not in the model		

Table 9: EMM – Day/Night*Low/High transmission interaction – overall test results – significant effects after sequential Sidak correction on $\alpha = 0.01$.

Call example	Wald Chi-Square	df	Significance
bf	956.009	3	0.000
bm1	Day/Night*Low/High was not in the model		
bm2	61.534	3	0.000
lskf	92.055	3	0.000
lskm1	Day/Night*Low/High was not in the model		
lskm2	Day/Night*Low/High was not in the model		
mp	412.639	3	0.000
trilH	65.953	3	0.000
trilL	Day/Night*Low/High was not in the model		
bittern	Day/Night*Low/High was not in the model		
kBoom	Day/Night*Low/High was not in the model		
kc	Day/Night*Low/High was not in the model		
weka	186.689	3	0.000
kaka	Day/Night*Low/High was not in the model		
hihi	Day/Night*Low/High was not in the model		
robin	Day/Night*Low/High was not in the model		
tui	Day/Night*Low/High was not in the model		
sad1	Day/Night*Low/High was not in the model		
sad2	Day/Night*Low/High was not in the model		
sad3	Day/Night*Low/High was not in the model		

Table 10: EMM – Open/Forest*Distance interaction – overall test results – significant effects after sequential Sidak correction on $\alpha = 0.01$.

**Call example**	**Wald Chi-Square**	**df**	**Significance**
bf	8550.861	9	0.000
bm1	2371.480	9	0.000
bm2	4058.628	9	0.000
lskf	3144.896	9	0.000
lskm1	3754.522	9	0.000
lskm2	2295.918	9	0.000
mp	6358.492	9	0.000
trilH	2486.788	9	0.000
trilL	760.840	9	0.000
bittern	Open/Forest*Distance was not in the model		
kBoom	Open/Forest*Distance was not in the model		
kc	Open/Forest*Distance was not in the model		
weka	5028.876	9	0.000
kaka	1723.986	9	0.000
hihi	564.019	9	0.000
robin	896.109	9	0.000
tui	1292.428	9	0.000
sad1	2344.956	9	0.000
sad2	1304.824	9	0.000
sad3	1632.038	9	0.000

S 4.2 Directionality Analysis

Table 11: EMM (Directionality Analysis) – Recorder direction – overall test results – significant effects after sequential Sidak correction on $\alpha = 0.01$

Call example	Wald Chi-Square	df	Significance
bf	14.200	3	0.003
bm1	24.095	3	0.000
bm2	15.940	3	0.001
lskf	21.661	3	0.000
lskm1	17.499	3	0.001
lskm2	20.604	3	0.000
mp	7.138	3	0.068
trilH	18.879	3	0.000
trilL	12.643	3	0.005
bittern	11.899	3	0.008
kBoom	5.538	3	0.136
kc	24.922	3	0.000
weka	18.863	3	0.000
kaka	21.825	3	0.000
hihi	28.167	3	0.000
robin	29.154	3	0.000
tui	19.779	3	0.000
sad1	20.599	3	0.000
sad2	30.529	3	0.000
sad3	19.396	3	0.000

S 4.3 Wind Speed Analysis

Table 12: EMM (Wind Speed Analysis) – Recorder direction – overall test results – significant effects after sequential Sidak correction on $\alpha = 0.01$

Call example	Wald Chi-Square	df	Significance
bf	23.979	3	0.000
bm1	24.474	3	0.000
bm2	17.195	3	0.001
lskf	13.472	3	0.004
lskm1	14.470	3	0.002
lskm2	15.239	3	0.002
mp	32.559	3	0.000
trilH	16.437	3	0.001
trilL	20.555	3	0.000
bittern	23.598	3	0.000
kBoom	7.587	3	0.055
kc	20.016	3	0.000
weka	20.853	3	0.000
kaka	37.682	3	0.000
hihi	32.495	3	0.000
robin	16.779	3	0.001
tui	30.388	3	0.000
sad1	17.507	3	0.001
sad2	63.109	3	0.000
sad3	24.694	3	0.000

Table 13: EMM (Wind Speed Analysis) – Wind level – individual test results – significant effects after sequential Sidak correction on $\alpha = 0.01$

Call example	Individual test results	Contrast Estimate	Std. Error	Wald Chi-Square	df	Significance
bf	moderate vs. calm	-0.094	0.006	246.735	1	0.000
	windy vs. moderate	-0.097	0.004	479.150	1	0.000
bm1	moderate vs. calm	-0.019	0.005	18.018	1	0.000
	windy vs. moderate	-0.032	0.004	63.340	1	0.000
bm2	moderate vs. calm	-0.032	0.005	46.148	1	0.000
	windy vs. moderate	-0.035	0.004	74.394	1	0.000
lskf	moderate vs. calm	-0.023	0.005	24.301	1	0.000
	windy vs. moderate	-0.036	0.004	81.429	1	0.000
lskm1	moderate vs. calm	-0.035	0.005	52.492	1	0.000
	windy vs. moderate	-0.041	0.004	94.754	1	0.000
lskm2	moderate vs. calm	-0.023	0.005	25.381	1	0.000
	windy vs. moderate	-0.030	0.004	51.035	1	0.000
mp	moderate vs. calm	-0.031	0.005	42.925	1	0.000
	windy vs. moderate	-0.049	0.004	149.117	1	0.000
trilH	moderate vs. calm	-0.015	0.005	10.758	1	0.001
	windy vs. moderate	-0.033	0.004	66.246	1	0.000
trilL	moderate vs. calm	0.002	0.004	0.131	1	0.717
	windy vs. moderate	-0.022	0.004	28.752	1	0.000
bittern	moderate vs. calm	-0.016	0.005	11.789	1	0.001
	windy vs. moderate	-0.001	0.004	0.048	1	0.827
kBoom	moderate vs. calm	-0.028	0.005	31.448	1	0.000
	windy vs. moderate	-0.011	0.005	5.909	1	0.015
kc	moderate vs. calm	0.000	0.004	0.001	1	0.978
	windy vs. moderate	-0.017	0.004	19.365	1	0.000
weka	moderate vs. calm	-0.045	0.005	81.488	1	0.000
	windy vs. moderate	-0.053	0.004	175.045	1	0.000
kaka	moderate vs. calm	-0.005	0.004	1.623	1	0.203
	windy vs. moderate	-0.030	0.003	80.155	1	0.000
hihi	moderate vs. calm	0.003	0.005	0.459	1	0.498
	windy vs. moderate	-0.032	0.004	73.159	1	0.000
robin	moderate vs. calm	-0.010	0.005	4.936	1	0.026
	windy vs. moderate	-0.025	0.004	41.229	1	0.000
tui	moderate vs. calm	-0.011	0.004	6.791	1	0.009
	windy vs. moderate	-0.032	0.004	67.643	1	0.000
sad1	moderate vs. calm	-0.010	0.005	4.861	1	0.027
	windy vs. moderate	-0.032	0.004	66.643	1	0.000
sad2	moderate vs. calm	0.000	0.005	0.001	1	0.981
	windy vs. moderate	-0.026	0.004	34.663	1	0.000
sad3	moderate vs. calm	-0.019	0.005	16.838	1	0.000
	windy vs. moderate	-0.029	0.004	54.368	1	0.000

Table 14: EMM (Wind Speed Analysis) – Wind level – overall test results – significant effects after sequential Sidak correction on $\alpha = 0.01$

Call example	Wald Chi-Square	df	Significance
bf	661.628	2	0.000
bm1	93.953	2	0.000
bm2	134.927	2	0.000
lskf	122.889	2	0.000
lskm1	162.494	2	0.000
lskm2	88.126	2	0.000
mp	221.131	2	0.000
trilH	87.483	2	0.000
trilL	29.810	2	0.000
bittern	14.525	2	0.001
kBoom	52.241	2	0.000
kc	20.499	2	0.000
weka	281.379	2	0.000
kaka	91.768	2	0.000
hihi	74.655	2	0.000
robin	51.044	2	0.000
tui	89.371	2	0.000
sad1	80.825	2	0.000
sad2	36.316	2	0.000
sad3	93.556	2	0.000

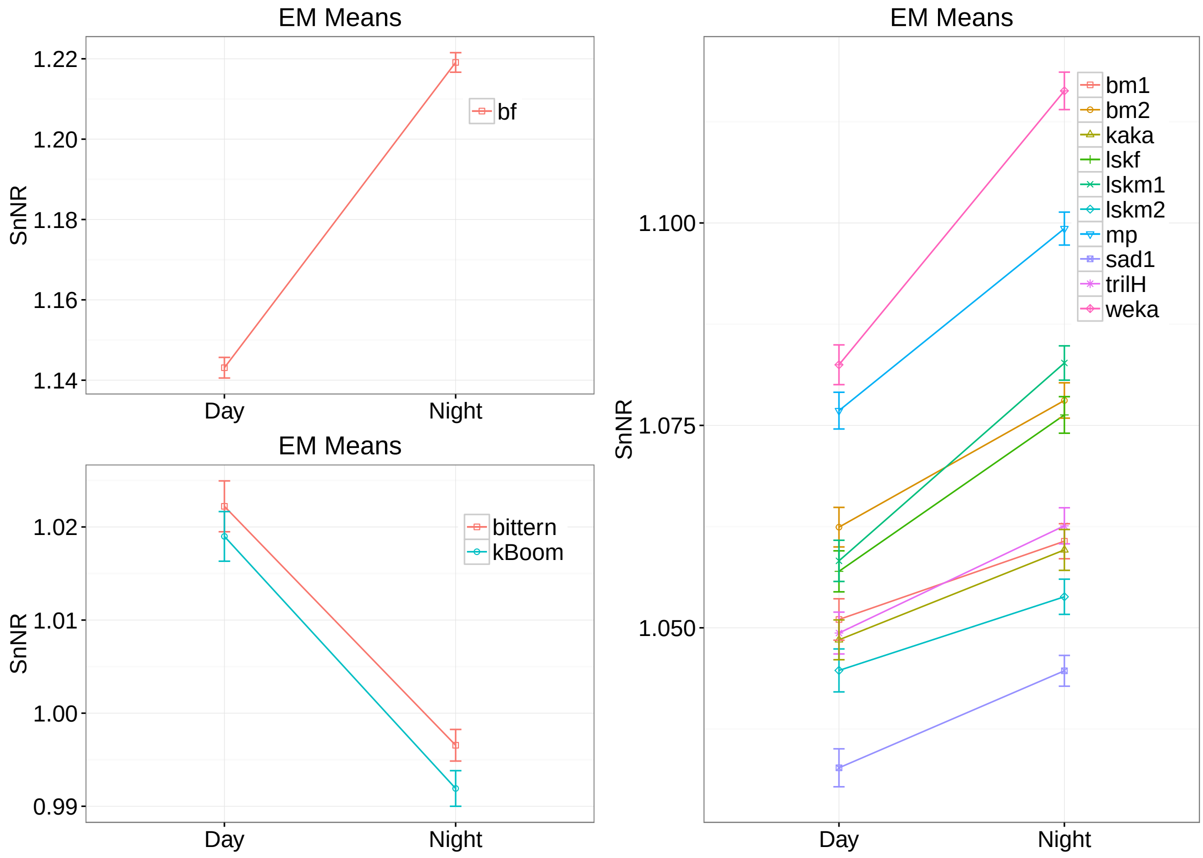

Figure 1: Estimated marginal means of SnNR for day vs night. Bars represent standard errors. Note that this figure was generated from 13 individual GLMs (for each bird sound example) and the lines were added to showcase the trend for each test result. bf=brown kiwi female, bm1=brown kiwi male example 1, bm2=brown kiwi male example 2, kBoom=kākāpō boom, lskf=little spotted kiwi female, lskm1=little spotted kiwi male example 1, lskm2=little spotted kiwi male example 2, mp=more-pork sound of morepork, sad1=saddleback example 1, and trilH=trill (high) sound of morepork.

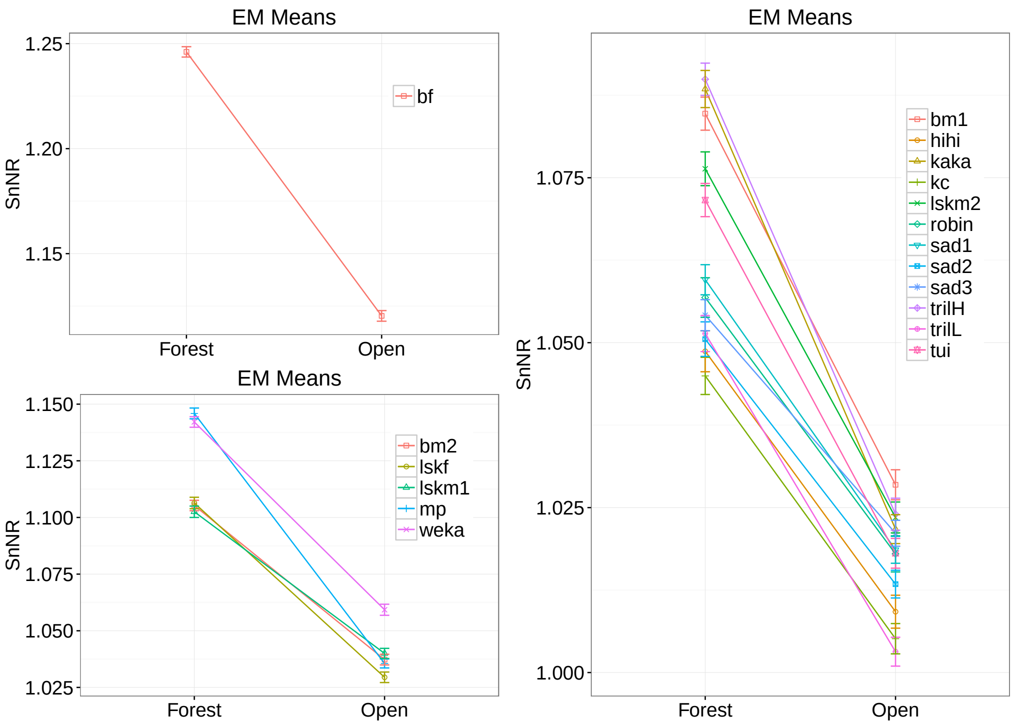

Figure 2: Estimated marginal means of SnNR for two different sites. Bars represent standard errors. Note that this figure was generated from 18 individual GLMs (for each bird sound example) and the lines were added to showcase the trend for each test result. bf=brown kiwi female, bm1=brown kiwi male example 1, bm2=brown kiwi male example 2, kBoom=kākāpō boom, kc=kakapo chinging, lskf=little spotted kiwi female, lskm1=little spotted kiwi male example 1, lskm2=little spotted kiwi male example 2, mp=more-pork sound of morepork, sad1=saddleback example 1, and trilH=trill (high) sound of morepork.

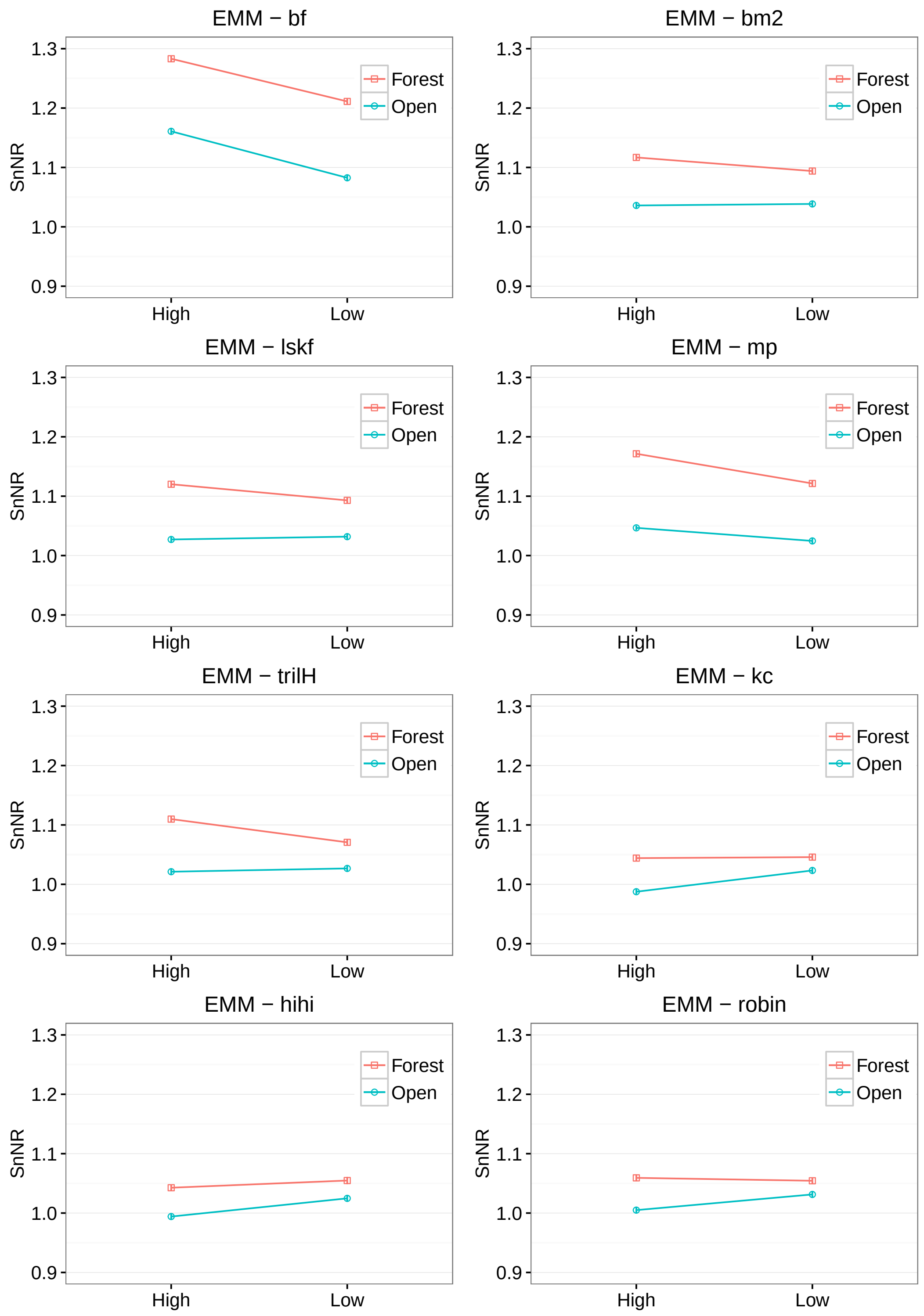

Figure 3: Estimated marginal means of SnNR and interaction effect of the transmission height and the habitat. Bars represent standard errors. Note that this figure was generated from 8 individual GLMs (for each bird sound example) and the lines were added to showcase the trend for each test result. bf=brown kiwi female, bm2=brown kiwi male example 2, lskf=little spotted kiwi female, mp=more-pork sound of morepork, trilH=trill (high) sound of morepork, and kc=kākāpō chinging.

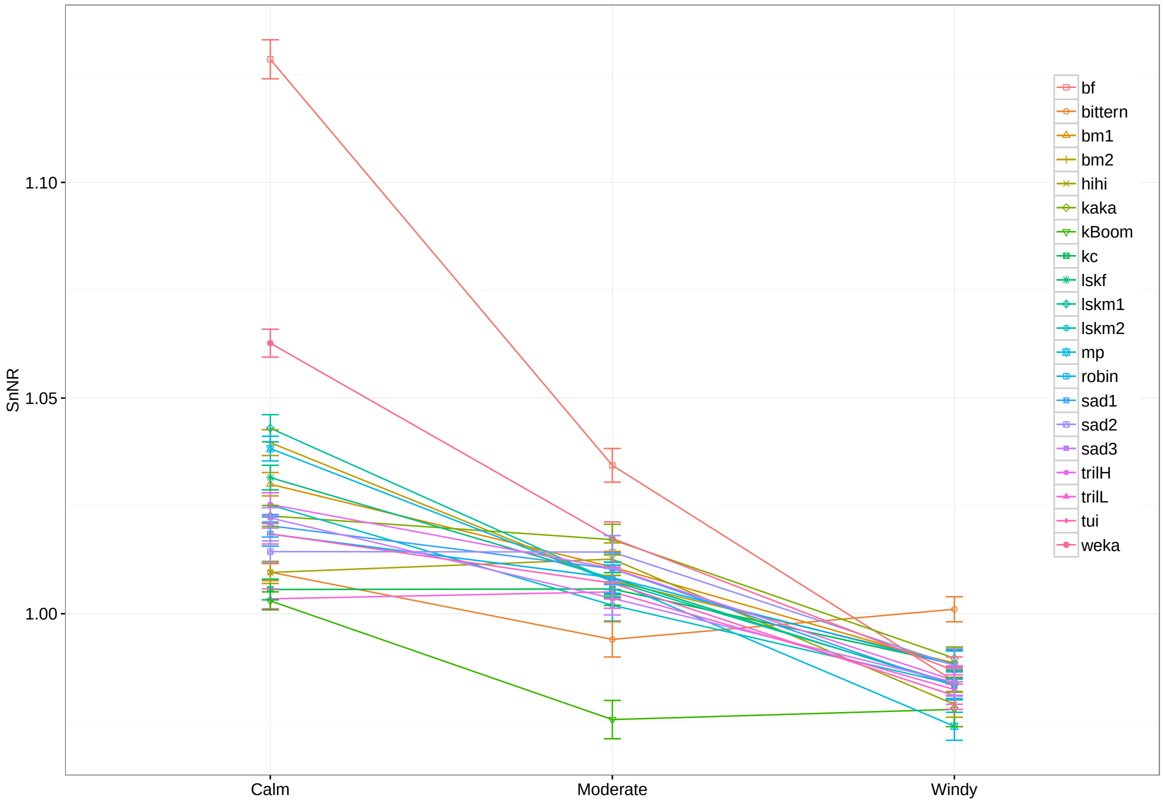

Figure 4: Estimated marginal means of SnNR against the different wind levels (Wind Speed Analysis). The lines were added to showcase the trend, otherwise the lines do not mean anything. bf=brown kiwi female, bm1=brown kiwi male example 1, bm2=brown kiwi male example 2, kBoom=kākāpō boom, kc=kākāpō chinging, lskf=little spotted kiwi female, lskm1=little spotted kiwi male example 1, lskm2=little spotted kiwi male example 2, mp=more-pork sound of morepork, sad1=saddleback example 1, sad2=saddleback example 2, sad3=saddleback example 3, trilH=trill (high) sound of morepork, and trilL=trill (low) sound of morepork.
